# Genomic signatures of dominant clones and evolutionary divergence of Acinetobacter baumannii in Thailand

**DOI:** 10.1099/mgen.0.001716

**Published:** 2026-05-14

**Authors:** Made Rai Dwitya Wiradiputra, Piyatip Khuntayaporn, Krit Thirapanmethee, Montri Yasawong, Mullika Traidej Chomnawang

**Affiliations:** 1 Antimicrobial Resistance Interdisciplinary Center (AMRIC), Faculty of Pharmacy, Mahidol University, Bangkok, Thailand; 2 Biopharmaceutical Sciences Program, Faculty of Pharmacy, Mahidol University, Bangkok, Thailand; 3 Department of Microbiology, Faculty of Pharmacy, Mahidol University, Bangkok, Thailand; 4 Program on Environmental Toxicology, Chulabhorn Graduate Institute, Bangkok, Thailand

**Keywords:** *Acinetobacter baumannii*, antimicrobial resistance, genomic surveillance, pathogen evolution

## Abstract

The global dissemination of carbapenem-resistant *Acinetobacter baumannii* (CRAB) is primarily driven by the expansion of two major international clones, IC1 and IC2, which have achieved widespread geographic distribution. Their success is further facilitated by the acquisition and dissemination of resistance determinants in healthcare settings. However, the evolutionary dynamics and genomic features of dominant clones in endemic settings such as Thailand remain insufficiently characterized. In this study, comparative genomic analyses were conducted on 650 *A. baumannii* genomes, including 38 newly sequenced CRAB isolates from tertiary hospitals across Thailand and 612 publicly available genomes, to examine lineage structure, resistome architecture and evolutionary trajectories. The dataset was dominated by ST2 (61.08%), followed by the emerging ST164 lineage (8.62%). Lineage-resolved analyses identified associations between STs and intrinsic resistance determinants, including enrichment of the AdeABC efflux component *adeC* and characteristic *blaADC/blaOXA-51-like* allelic combinations within dominant clones. Acquired resistance genes were widespread, with *blaOXA-23-like* carbapenemases predominating across lineages. Phylogenomic reconstruction showed that ST164 [international clone 11 (IC11)] occupies a distinct evolutionary position relative to established epidemic clones. Bayesian phylodynamic inference estimated a recent emergence of ST164 in Thailand around 2013–2014, with rapid initial clonal expansion followed by a period of relative population stability. Overall, this study reveals lineage-associated resistome features and distinct evolutionary trajectories among dominant and emerging *A. baumannii* lineages in Thailand, providing the value of genome-scale surveillance and lineage-resolved analyses for monitoring high-risk clones in endemic settings.

**Impact Statement**

Carbapenem-resistant *Acinetobacter baumannii* (CRAB) surveillance has traditionally emphasized acquired resistance genes and sequence type assignment, often overlooking lineage-resolved intrinsic resistome variation and evolutionary context. By integrating newly sequenced clinical isolates with a large collection of publicly available genomes, this study establishes a population-scale genomic framework for understanding CRAB dissemination in Thailand. The results demonstrate that dominant lineages are characterized by distinct intrinsic resistance signatures, including lineage-associated *blaADC/blaOXA-51-like* allelic combinations and differential presence of the AdeABC efflux component *adeC*, features that are not typically captured by conventional resistance gene profiling alone. In addition, genome-scale evolutionary analyses reveal that emerging lineages can follow evolutionary trajectories that are distinct from established epidemic clones, highlighting the importance of incorporating phylogenomic and temporal perspective into epidemiological surveillance. Collectively, these findings highlight the value of allele-level intrinsic resistome profiling and evolutionary inference as complementary tools of resistance evolution in settings with sustained CRAB transmission.

## Data Summary

All genomic data of newly sequenced isolates were deposited in the NCBI GenBank database under the BioProject accession PRJNA854605 and PRJNA1419516. All supporting data are provided in the supplementary files. All accession numbers of analysed genomes are given in Table S3.

## Introduction

*Acinetobacter baumannii* epitomizes successful pathogen evolution, as selective pressures associated with niche adaptation have driven a few dominant lineages to acquire virulence and resistance determinants that confer enhanced fitness in clinical environments [1,2]. Of particular concern is the rapid emergence of carbapenem-resistant *A. baumannii* (CRAB) that become an important nosocomial pathogen and frequently displays multidrug-resistant (MDR) or even extensively drug-resistant (XDR) phenotypes, which represents a critical public health threat [3,4]. CRAB is recognized as one of the deadliest bacterial pathogens [5,6], with resistance rates consistently exceeding those of other priority pathogens [7]. On average, the resistance rates to carbapenems range between 40 and 90%. The expanding spectrum of resistance and international spread of CRAB have also prompted the World Health Organization (WHO) to maintain its classification as a critical priority pathogen in the global framework for research and development of intervention strategies to address the antimicrobial resistance crisis [8,9].

Advances in genomic epidemiology have greatly enhanced our understanding of the global dissemination and clonal dynamics of CRAB. Whole-genome sequencing and phylogenetic analyses have revealed that the worldwide spread of CRAB has been largely attributed to the expansion of two major international clones, IC1 and IC2. The progression of antimicrobial resistance within CRAB populations is further facilitated by the acquisition and dissemination of resistance genes among epidemic clones, often mediated by mobile genetic elements, such as ISAba-type insertions, transposons and resistance islands carrying multiple resistance genes [10,12]. This has resulted in an increased prevalence of resistance genes among clinical CRAB, exemplified by the substantial rise in acquired carbapenemases from 62.8% before 2010 to 96% after 2010, with *blaOXA-23-like* genes now predominating [13]. In parallel, several emerging or regionally restricted ICs have also been described in recent years [13,15]. This ongoing clonal diversification can occur at different rates or through mechanisms distinct from those in the initial clones [16,18], continually reshaping CRAB population structure and influencing both the resistome and the distribution of dominant clones. Such a comprehensive understanding of these evolutionary dynamics at both global and regional scales is crucial for anticipating shifts in resistance patterns and their clinical implications.

In Thailand, antimicrobial susceptibility of clinical *A. baumannii* isolates has been tracked by the National Antimicrobial Resistance Surveillance Center (NARST). Annual antibiogram reports indicate a dramatic increase in carbapenem resistance among the *A. calcoaceticus-baumannii* complex, rising from ~5% in 2000 to 70–80% during 2019–2024 [19,20]. Consistent with this trend, resistance rates of CRAB in Thailand are among the highest in the Asia-Pacific region [21]. Previous studies further indicate that the CRAB population in the country is predominated by ST2 (IC2), with ST164 (IC11) reported as the second-most common lineage and *blaOXA-23-like* as the most prevalent carbapenemase gene [22,24].

Moreover, several studies have characterized resistance genes associated with mobile genetic elements, offering deeper insights into the potential dissemination of resistance within *A. baumannii* population [25,27]. However, most studies to date have relied on traditional genotyping approaches, or their genomic analysis has been limited to single regions or hospital networks. Consequently, despite the increasing availability of public genomes, comprehensive analyses addressing evolutionary dynamics and lineage-resolved genomic architecture underlying the dissemination and diversification of CRAB across Thailand remain underexplored.

In this study, we analysed a comprehensive dataset comprising clinical CRAB isolates collected across tertiary hospitals in Thailand in recent years, together with publicly available *A. baumannii* genomes (*n*=650), through a comparative genomic approach. Our findings highlight the clonal architecture and lineage-specific patterns of several resistance genes, including the characteristic distribution of intrinsic *blaOXA-51-like* and *blaADC* allelic variants among high-risk clones circulating in Thailand. Phylodynamic analyses further revealed distinct evolutionary trajectories between the predominant ST2 and emerging ST164, supporting recent emergence of ST164.

## Methods

### Bacterial isolates

CRAB isolates from a laboratory collection were selected based on their reported antimicrobial susceptibility, geographic origin and clonal relatedness. These isolates had been obtained from hospitals across Thailand. Preliminary species identification at the collection sites was performed using automated microbial identification system. Further confirmation in the laboratory was conducted through Gram staining, amplification of the intrinsic *blaOXA-51* gene and multi-locus sequence typing (MLST) based on Pasteur scheme.

### Genomic DNA extraction and whole-genome sequencing

Mid-log phase cultures were used for genomic DNA extraction following the standard protocol of Gentra Puregene Yeast/Bact Kit (Qiagen, Germany), with the exception that centrifugation after isopropanol precipitation step was performed at 8,000 r.p.m. DNA quality and concentration were assessed using a NanoDrop nucleic acid analyser (Hercuvan Lab System, UK), QFX fluorometer (DeNovix, USA) and 1% agarose gel electrophoresis.

For long-read sequencing, DNA libraries were prepared using ONT Rapid Barcoding Kit 24 V14 (SQK-RBK114.24) and loaded into a MinION R10.4.1 flow cell (Oxford Nanopore Technologies, UK). Sequencing was carried out on a MinION Mk1c device for 24 h. Basecalling was performed using Dorado v.0.7.3 (https://github.com/nanoporetech/dorado) with a super accuracy model. For a subset of selected isolates, short-read sequencing was performed either on the Illumina NextSeq 500 (PE150) or NovaSeq 6000 (PE150) platform (Illumina, USA). FASTQ files generated from both Nanopore and Illumina sequencing were used for subsequent bioinformatics analysis as described below.

### *De novo* genome assembly and annotation

Illumina and Nanopore sequence data were initially screened for potential contamination by mapping against the Standard-8 database using Kraken2 [28]. For Nanopore reads, adapter trimming and quality filtering were carried out using Porechop v.0.2.4 (https://github.com/rrwick/Porechop) and Filtlong v.0.2.1 (https://github.com/rrwick/Filtlong), with parameters set to a minimum read length of 1,000 bp and a minimum mean Phred quality score of 10. Illumina reads were processed using Fastp v.0.24.0 [29] in default parameters for adapter trimming and quality filtering.

*De novo* genome assembly was conducted through a decision-making workflow based on the availability of short-read data and estimated long-read coverage. When both Nanopore long reads and Illumina short reads were available, hybrid assembly was conducted using the short-read first, long-read bridge approach in Unicycler v.0.5.1 [30]. If the resulting assembly was highly fragmented, an alternative long-read-first, short-read polishing approach was employed. In this approach, a preliminary long-read assembly was generated using Flye v.2.9.3 [31] followed by Medaka v.2.0.0 (https://github.com/nanoporetech/medaka), which was then used as input for hybrid assembly in Unicycler v.0.5.1.

For isolates with only Nanopore long-read available, sequencing coverage was first estimated assuming an average *A. baumannii* genome size of 4 Mb. If the estimated coverage was ≥70×, a high-confidence consensus assembly was performed using Autocycler v.0.5.1 (https://github.com/rrwick/Autocycler). In this pipeline, reads were subsampled into four sets with coverage ranging from 30 to 70× depending on the initial coverage. Each subsample was assembled independently using multiple long-read assemblers, including Flye v.2.6, NECAT v.0.0.1 and Raven v.1.8.3. The resulting contigs were compressed into a compacted De Bruijn graph, clustered into putative replicons, trimmed and resolved to generate a consensus assembly. If the estimated long-read sequencing coverage was below 70, assembly was performed using Flye v.2.9.3 [31] alone. Assemblies produced by both Autocycler and Flye were polished using Medaka v.2.0.0. Structural and functional annotations were performed using Prokka v.1.14.6 [32] and the NCBI prokaryotic genome annotation pipeline [33]. Assembly quality was evaluated using QUAST v.5.3.0 [34] and CheckM v.1.2.4 [35]. Assembly graphs are visualized using Bandage v.0.9.0 [36].

### Dataset of publicly available *A. baumannii* genomes

A comprehensive genome dataset was compiled, comprising isolates sequenced in this study and selected publicly available *A. baumannii* genomes as of 31 January 2025. Public genomes were retrieved from Pathogenwatch and NCBI GenBank, filtered by geographic origin (Thailand). Metadata and assembly files from Pathogenwatch were retrieved from its web server (https://pathogen.watch/), while NCBI GenBank data were downloaded using the NCBI Datasets command-line tool (https://www.ncbi.nlm.nih.gov/datasets/). For entries with only raw sequencing reads available, short-read assembly was performed using a standard pipeline: reads quality-filtered with Fastp v.0.24.0 [29] and assembled using Shovill v.1.1.0 (https://github.com/tseemann/shovill). Publicly available genomes were manually curated to eliminate redundancy and were evaluated based on quality criteria such as average nucleotide identity (ANI), GC% and genome size.

### Identification of sequence types and capsular polysaccharide loci

A Perl-based MLST script (https://github.com/tseemann/mlst) was used to screen assembled contigs and assign sequence types (STs) based on the Pasteur scheme for *A. baumannii* available in PubMLST database (https://pubmlst.org/). The MLST profiles were used to infer population structure and identify clonal complexes using the global optimal eBURST algorithm implemented in PHYLOViZ v.2.0 [37]. Capsular polysaccharide locus typing was performed using Kaptive based on the *A. baumannii* K and OC locus databases [38].

### Resistome profiling

ABRicate (https://github.com/tseemann/abricate) was employed to examine the contigs and detect antimicrobial resistance genes. Identification of acquired resistance genes was performed using the NCBI AMRFinderPlus [39] and ResFinder [40] databases. The CARD database [41] was used specifically to detect intrinsic resistance genes, such as those related to efflux pumps and outer membrane proteins.

### Pangenome and phylogenomic reconstruction

All genomes in the final dataset were annotated using Prokka, generating annotated genomes in GFF format. Core genome alignment was constructed across all genomes in the dataset using Roary v.3.13.0 [42]. Prior to phylogenomic analysis, duplicate (identical) core genome sequences were identified and excluded from the Roary core genome alignment using SeqKit v.2.8.2 [43]. The population structure phylogeny of the final dataset was inferred using maximum-likelihood approach in IQ-TREE2 v.2.3.6 [44], employing automated nucleotide substitution model selection (TEST), 1,000 ultrafast bootstrap replicates and 1,000 SH-like approximate likelihood ratio test replicates. The final tree was visualized using iTOL [45].

For lineage specific-phylogenomic analyses, recombination detection and masking were performed using ClonalFrameML v.1.13 [46] with a transition/transversion ratio (kappa) of 2.0, applied independently per lineage. A maximum-likelihood starting tree, required as input for ClonalFrameML, was first constructed for each lineage using IQ-TREE2 with the GTR+G substitution model and fast tree search. Recombination-masked SNP alignments were subsequently extracted using SNP-sites v.2.5.1 [47] and used to reconstruct final lineage-specific maximum-likelihood phylogenies using IQ-TREE2 with the same parameters as the entire dataset.

### Temporal analysis of ST164

Bayesian temporal analysis was performed using the ST164 clone dataset. Temporal signal was first assessed by calculating the coefficient of determination (*R*^2^) through linear regression of root-to-tip genetic distance against sampling time in TempEst v.1.5.3 [48], based on a maximum-likelihood phylogenetic tree inferred from IQ-TREE2. Recombination-masked SNP alignment was extracted using SNP-sites retaining only complete columns, with constant site counts provided for ascertainment bias correction prior to temporal analysis. The recombination-masked core genome SNP alignment and corresponding temporal metadata were used to generate an XML configuration file in BEAUti v.2.7.8 [49]. Bayesian evolutionary analyses were then conducted in BEAST v.2.7.6 [49], generating a posterior distribution of phylogenetic trees according to the specified configuration. Multiple molecular clock models (strict, relaxed lognormal and random local) were tested, with each model run for 50 million Markov chain Monte Carlo (MCMC) steps and sampling every 1,000 steps. The final selected model was run for 100 million MCMC steps to ensure adequate convergence. Maximum clade credibility (MCC) trees were summarized from the resulting tree sets using TreeAnnotator v.2.7.6 [49] with a 10% burn-in and mean node heights. Model performance was assessed based on convergence, effective sample size (ESS) and mean posterior-likelihood values in Tracer v.1.7.7 [50]. Bayesian Skyline reconstructions were also performed using Tracer to predict changes in effective population size over time.

## Results

### Clinical and phenotypic characteristics of CRAB isolates selected for whole-genome sequencing

The present study included CRAB isolates collected from two independent surveillance periods for whole-genome sequencing. The first set (*n*=17) originated from a nationwide study conducted between 2016 and 2017, comprising 135 clinical CRAB isolates [22]. The second set (*n*=21) was part of a larger study involving 317 clinical CRAB isolates (unpublished data). Among 452 isolates collected from both cohorts, ST2 was the most predominant lineage. Other major lineages were also observed, including ST164, ST16 and ST25, which together with ST2 constituted ~90% of the isolates (Fig. S1, available in the online Supplementary Material). Based on this collection, a total of 38 clinical CRAB isolates from both studies were selected to represent broader geographical regions, diverse genetic lineages (as determined by pre-WGS MLST) and varying AMR phenotypes. A summary of the selected 38 CRAB and their clinical characteristics is provided in Table S1.

All isolates had been initially identified and tested for antimicrobial susceptibility at the respective hospitals using biochemical-based methods and automated systems. Upon arrival at the laboratory, molecular confirmation of *A. baumannii* species was performed by PCR targeting the intrinsic *blaOXA-51* gene [51]. Further species verification and ST assignment were carried out by amplifying and sequencing the seven housekeeping genes included in Pasteur MLST scheme [52]. Among CRAB isolates included in this study, the majority (76%) were recovered from respiratory specimens, particularly sputum and tracheal aspirates, followed by urine and pus samples. Isolates originated from nine provinces across five regions of Thailand, with the northern and northeastern regions contributing the largest proportions. ST2 was the most prevalent lineage, accounting for 68% (26/38) of the isolates. Other represented STs included ST25, ST16, ST52, ST109 and ST164, reflecting genetic diversity among the selected isolates. Based on AST, 33 isolates (86%) exhibited XDR phenotypes, while the remaining five were classified as MDR. A detailed antibiogram of these isolates is provided in Table S1.

### Lineage-associated resistome profile among newly sequenced CRAB isolates

**Fig. 1.**
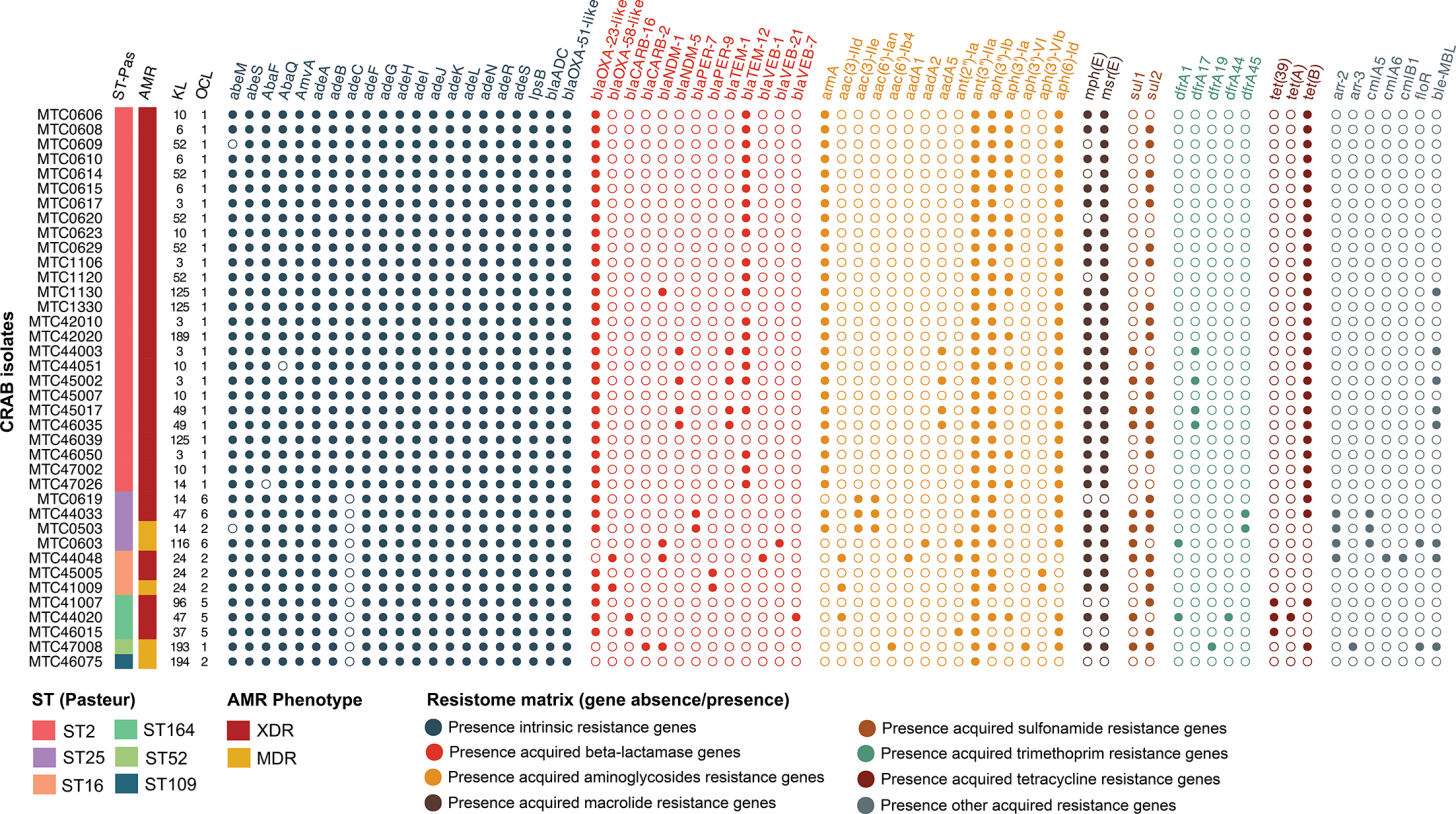
Resistome profile of 38 sequenced CRAB isolates. Presence-absence matrix of AMR genes identified in each isolate, grouped by functional class. Isolates are ordered by ST, with AMR phenotype, K locus (KL) and outer core locus (OCL) shown on the left. Gene detection was based on results from CARD and NCBI AMRFinder databases, using 80% identity and coverage cutoffs.

Characterization of the resistome revealed variability among the 38 sequenced CRAB isolates in this study, with differences observed across STs, especially in the acquired resistance genes. The resistome matrix (Fig. 1) highlights a distinct cluster of intrinsic resistance genes, alongside multiple gene clusters typically associated with acquired resistance. Within the intrinsic gene cluster, ST2 isolates were uniquely characterised by the presence of a complete *adeABC* efflux operon, whereas other STs generally lacked *adeC*. This pattern remained consistent when coverage and identity thresholds were relaxed to 60%, except for one ST25 isolate (MTC0603). Furthermore, characteristic allele combinations of *blaADC* and *blaOXA-51-like* genes were observed across several STs: ST2 carried *blaADC-73* and *blaOXA-66*, ST25 carried *blaADC-26* and *blaOXA-64* and ST16 carried *blaADC-169* and *blaOXA-402* (Table S2). Minor variations within ST2 were also noted, with one isolate each lacking *abeM*, *abaF* or *abaQ*.

While intrinsic resistance determinants were relatively conserved, the presence and absence of acquired resistance genes varied across CRAB isolates, with distinct patterns also observed between STs (Fig. 1). Notably, XDR isolates generally harboured a broader range of acquired resistance genes, yet there was no strict one-to-one correlation between gene count and phenotypic resistance category. Most isolates (*n*=34) carried the *blaOXA-23* gene, a class D *β*-lactamase commonly associated with carbapenem resistance in *A. baumannii*. No acquired *β*-lactamase genes from class C were detected; only class A (*blaCARB*, *blaPER*, *blaTEM* and *blaVEB*), class B (*blaNDM*) and class D (*blaOXA-23*, *blaOXA-58*) enzymes were identified.

The resistome appeared more conserved among ST2 isolates compared to other STs. The *blaTEM* gene was nearly exclusive to ST2, absent in only two isolates, represented by either TEM-1 or TEM-12 allelic variants. Notably, four ST2 isolates carried *blaNDM-*5. All ST2 isolates also carried the 16S rRNA methyltransferase *armA* and the tetracycline resistance gene *tet(B*). Aminoglycoside-modifying enzymes (AMEs) such as *ant(3″)-IIa*, *aph(3″)-Ib* and *aph(6)-Id* were highly prevalent in ST2 isolates, while a greater diversity of these enzymes was observed in non-ST2 genomes. Additionally, class A *β*-lactamase *blaCARB*, *blaPER* and *blaVEB* were sporadically detected in isolates belonging to ST16, ST25, ST52 and ST164.

### Population-scale resistome architecture of *A. baumannii* genomes from Thailand

**Fig. 2.**
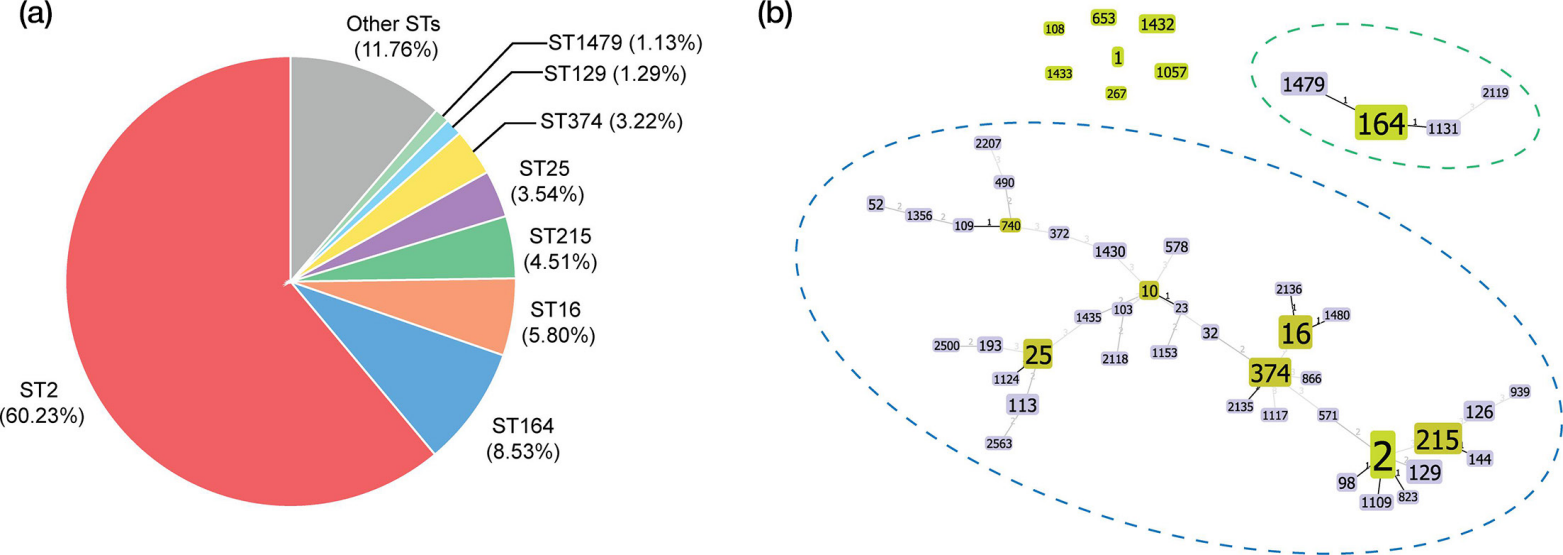
Lineage distribution and population structure of *Acinetobacter baumanni* genomes from Thailand. (**a**) Proportional distribution of major STs based on Pasteur MLST scheme. (**b**) Minimum-spanning tree of MLST alleles generated using the goeBURST algorithm. The dominant lineage grouping is highlighted by the blue dashed outline and ST164-associated lineage is marked by the green dashed outline.

To contextualize these findings within a broader epidemiological framework of *A. baumannii* resistome in Thailand, a total of 650 genomes comprising both sequenced CRAB isolates in this study (*n*=38) and publicly available non-redundant genomes (*n*=612) were analysed (Table S3). The *A. baumannii* public genomes from Pathogenwatch and NCBI GenBank databases were filtered based on geographic origin (Thailand), with quality control criteria including GC range (38.5–41.5%), genome size (3.2–4.6 Mb), reflecting the expected genomic characteristics of *A. baumannii* in GenBank, and ANI (>97%). As shown in Fig. 2(a), the largest proportion of isolates belonged to ST2, with other major STs including ST164, ST16, ST215, ST25, ST374, ST129 and ST1479. Collectively, these major STs accounted for nearly 90% of the genomes analysed. The remaining ~11% of isolates comprised a diverse array of less common STs, many of which were represented by a single isolate. The minimum-spanning tree based on MLST allelic profiles revealed two major lineage groupings within the dataset (Fig. 2b), providing additional context for the lineage distribution. This lineage structure was examined in greater detail using genome-scale analyses in subsequent sections.

**Fig. 3.**
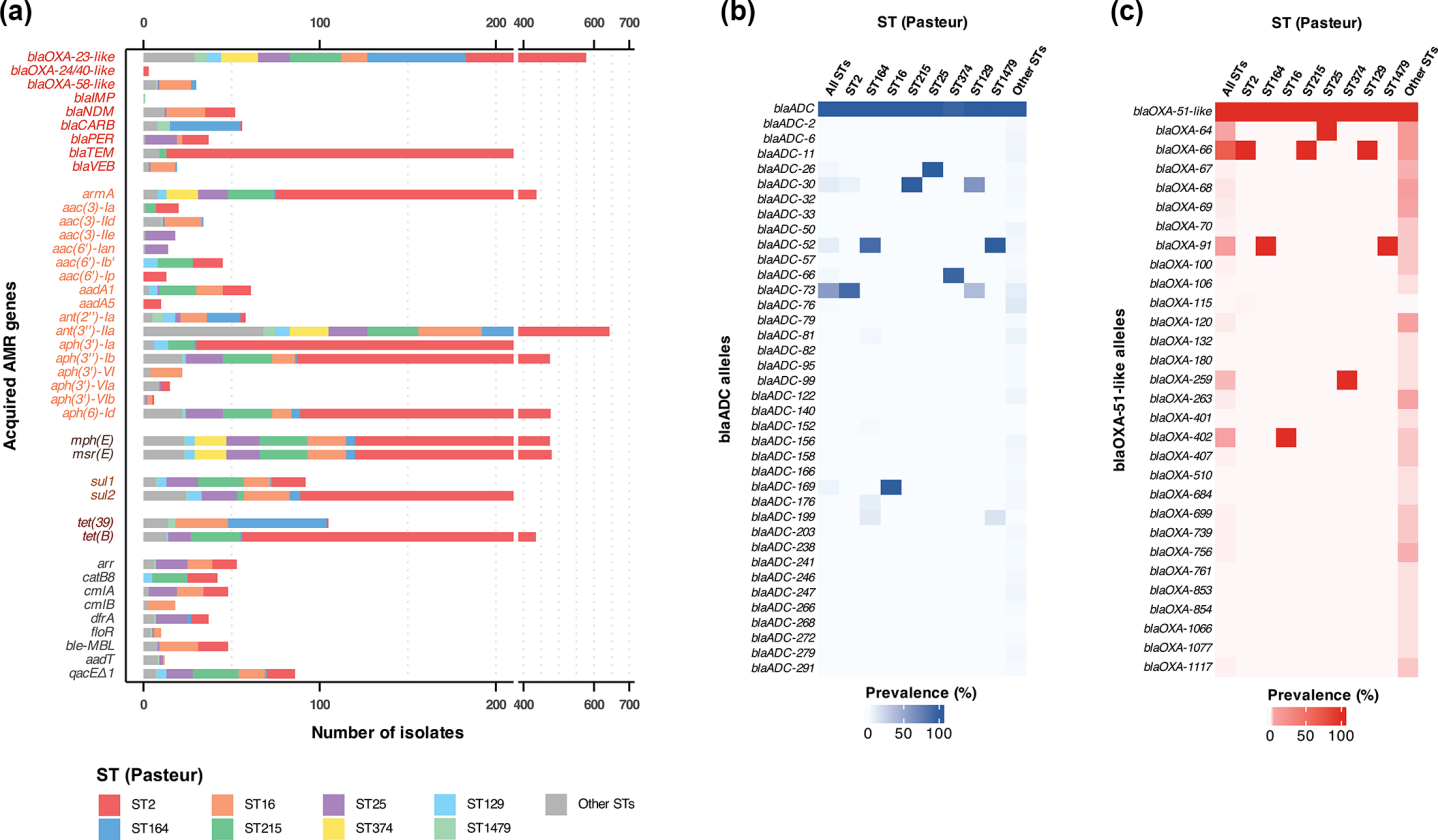
Resistome characterization of 650 *A. baumannii* genomes from Thailand. (**a**) Prevalence of acquired and intrinsic antimicrobial resistance (AMR) genes across 650 genomes. Stacked horizontal bar plots show the number of genomes carrying each AMR gene, stratified by major STs (ST2, ST164, ST16, ST215, ST25, ST374, ST129 and ST1479) and a combined group of less common STs (‘Other STs’). Genes are grouped by functional class: intrinsic resistome excluding intrinsic *β*-lactamases (grey text), acquired *β*-lactamases (red), aminoglycoside resistance determinants (orange), macrolide resistance genes (purple), sulphonamide resistance genes (brown), tetracycline resistance genes (blue) and other resistance mechanisms (green). (**b, c**) Distribution of *blaADC* (**b**) and *blaOXA-51-like* (**c**) alleles across 650 genomes. Heatmaps showing the presence of individual *blaADC* (**a**) and *blaOXA-51-like* (**b**) alleles across genomes, grouped by ST based on the Pasteur MLST scheme. Each row represents a detected allele, and each column corresponds to a distinct ST. Colour intensity reflects the percentage of genomes within each ST that carry the respective allele.

The overall profile of intrinsic resistance genes remained largely consistent between the 38 newly sequenced CRAB isolates and the expanded dataset of 650 genomes. In addition, the inclusion of a broader diversity of STs revealed several notable patterns. Most prominently, the distribution of the efflux component *adeC* further supported the strong association of this gene with ST2 and its closely related variants. Among the 650 genomes, *adeC* was detected in 69.38% of isolates, making it the least prevalent intrinsic resistance gene in the dataset, whereas other intrinsic genes were present in at least 97% of genomes (Fig. 3a). When analysed by ST, *adeC* was found in all ST2 and ST215 genomes, while its prevalence in ST129 was 85.75%. On the other hand, prevalence of *adeC* in STs other than ST2, ST129 and ST215 was below 10%, with most detections occurring in genomes belonging to single-locus variants (SLVs) or double-locus variants (DLVs) of ST2 (e.g. ST98, ST571 and ST1109). *AbaF* was absent in 4% (*n*=26) of genomes, with most of them belonging to ST2. In addition, *AmvA*, another efflux pump gene, was absent in most ST113, contributing to its lower prevalence in that lineage, with only 1 out of 5 ST113 genomes harbouring the gene (Table S4). Examination of the efflux pump regulator genes (*adeR*, *adeS*, *adeL* and *adeN*) across all 650 genomes revealed high prevalence (>97%) of all four regulators. Non-synonymous SNP analysis relative to the corresponding sequences in *A. baumannii* ATCC19606 (GenBank Accession CP045110.1) showed predominantly lineage-specific substitution patterns fixed within individual STs, with the exception of *adeL*, for which over 85% of genomes shared identical sequences with *A. baumannii* ATCC19606 (Figs S2–S5).

A similar lineage-structured pattern was also observed for intrinsic *blaADC* and *blaOXA-51-like* alleles in the expanded dataset with genomes within the same ST typically carrying the same allele. As of September 2025, the Beta-Lactamase Database (BLDB) lists 293 *blaADC* and 396 *bla-OXA51-like* allelic variants [53]. Across the 650 genomes analysed, 37 *blaADC* and 29 *blaOXA-51-like* alleles were identified, with *blaADC-73* and *blaOXA-66* being the most prevalent (Fig. 3b, c). Within-lineage allele diversity was more frequently observed for *blaOXA-51-like*, whereas *blaADC* alleles were generally conserved within an ST. Although individual alleles from each gene family were shared across the same lineages, the combined *blaADC/blaOXA-51-like* allele profiles were largely lineage-associated, with only limited variation.

A broader view of the acquired resistance genes among the 650 *A*. *baumannii* genomes further highlighted notable diversity, with some of the genes prominently more prevalent in specific STs (Fig. 3a). Among the acquired *β*-lactamase genes, *blaOXA-23-like* was the most widespread, identified in 577 genomes (88.77%), predominantly among ST2 isolates. Most major lineages (ST2, ST164, ST215, ST25, ST374, ST129 and ST1479) showed high prevalence of this gene, while ST16 and those grouped as ‘Other STs’ exhibited much lower detection rates, typically around 40% (Table S4). The second most prevalent in this group was *blaTEM*, with 92.86% of *blaTEM*-positive genomes belonging to ST2. Other *β*-lactamase genes such as *blaCARB*, *blaPER*, *blaVEB*, *blaNDM* and *blaOXA-58-like* were less frequently detected and distributed broadly across STs. The *blaOXA-24/40-like* family and *blaIMP* were the rarest, detected in only a single genome from ST1479, respectively. Interestingly, *blaVEB*, *blaNDM* and *blaOXA-58-like* were more common in ST16 than in other lineages, while *blaCARB* was more frequently observed in ST164 and ST1479.

Aminoglycoside resistance genes were widely distributed, with *ant(3″)-IIa*, *aph(3″)-Ib* and *aph(6)-Id* being the most frequently detected aminoglycoside-modifying enzymes (AMEs), largely represented in ST2 genomes. Among these, *ant(3″)-IIa* was found in 99.08% of all genomes. The 16S rRNA methyltransferase gene *armA* was primarily identified in ST2, ST215, ST25, ST374 and ST129 but occurred at much lower frequencies in other STs. Compared to the more conserved AME gene profile in ST2, a broader diversity of aminoglycoside resistance genes was observed among non-ST2 lineages.

Other functional classes of acquired resistance determinants were also well represented across the dataset. Macrolide resistance genes *mph(E*) and *msr(E*) were prevalent in the five most dominant STs, though their frequency was notably lower in ST164 (~14%). The sulphonamide resistance gene *sul2* was detected in nearly 60% of the genomes, with high prevalence (>70%) in ST2, ST16, ST25 and ST129. Conversely, *sul1* is generally less common yet exhibited higher prevalence in ST25 and ST129. Trimethoprim resistance genes (*dfrA* variants) were rare, carried only in 5% of the genomes. Among the tetracycline resistance genes, *tet(B*) was the most common and predominantly associated with ST2 and ST215, while *tet(39*) was more frequently detected in ST16 and ST164. Collectively, the lineage-associated patterns observed across both intrinsic and acquired resistance determinants across 650 genomes are in line with the notion of clonal expansion and dissemination of dominant lineages, most notably ST2, across Thailand.

### Phylogenomic reconstruction reveals distinct evolutionary trajectories of predominant *A. baumannii* ST2 and emerging ST164 in Thailand

To infer the clonal relatedness and evolutionary trajectories of *A. baumannii* circulating in Thailand, a phylogenomic analysis was performed based on core-genome alignment. Initially, the pangenome structure was constructed to identify gene clusters classified as part of the core genome, defined as those present in ≥99% of the 650 *A*. *baumannii* genomes analysed (Table S3). However, 29 genomes were found to share identical core genome sequences to other isolates in the dataset, predominantly pairs of genomes from the same geographic region, suggestive of localized transmission rather than a single large outbreak event. To avoid distortion of phylogenomic resolution, these genomes were excluded from subsequent analysis. The final dataset comprised 621 genomes. Importantly, the distribution of STs remained well represented after this filtering, ensuring the diversity necessary to capture the broader epidemiological landscape of *A. baumannii* in Thailand.

**Fig. 4.**
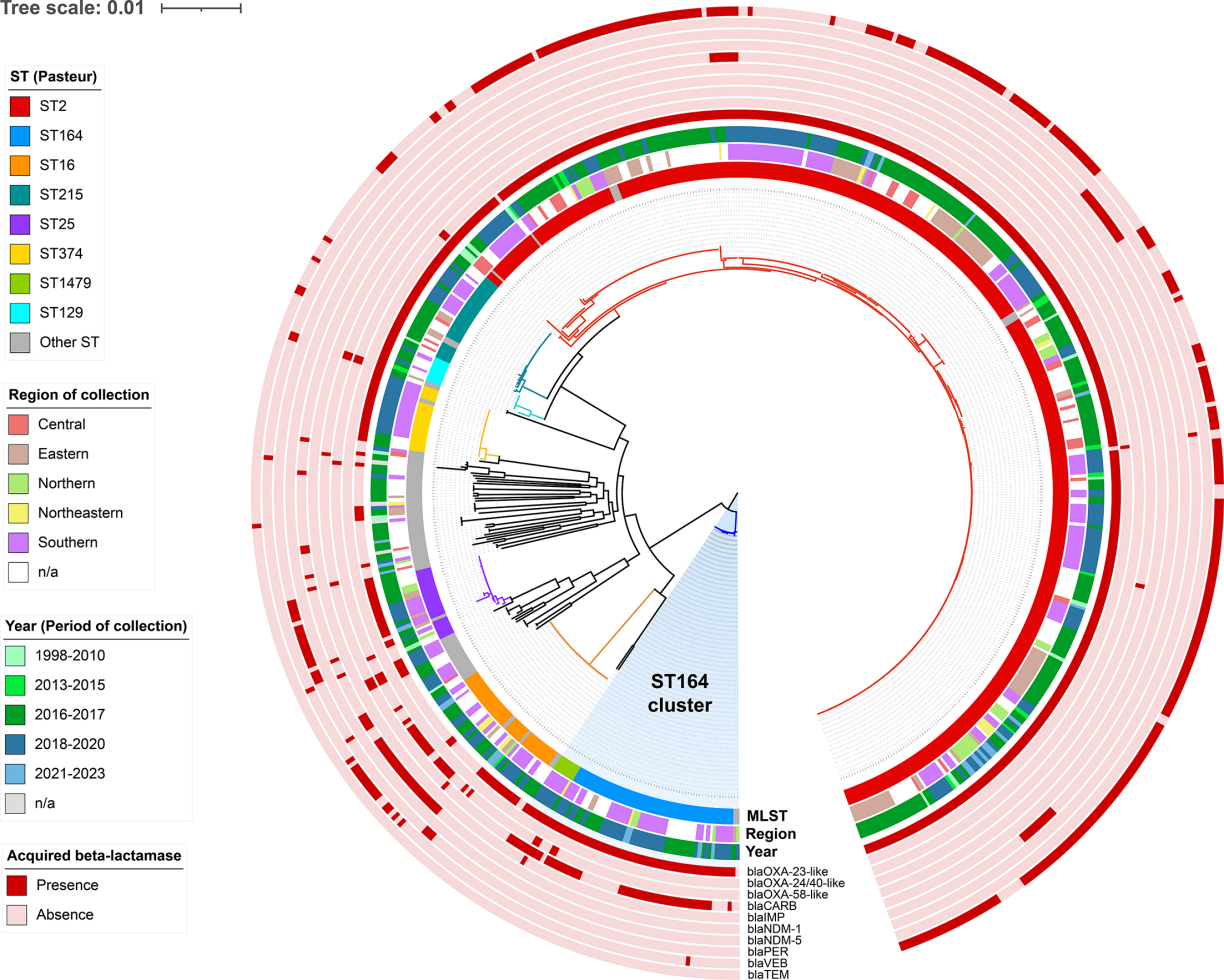
Maximum-likelihood phylogeny of 621 *A*. *baumannii* genomes originating from Thailand. The tree was inferred based on core genome alignment across 2,109 gene clusters. Phylogenetic tree was reconstructed using IQ-TREE2 under GTR+F+I+G4 substitution model with 1,000 ultrafast bootstrap replicates. The final tree was visualized in iTOL and displayed with unrooted topology. Coloured branches represent major STs and their variants, while concentric rings indicate metadata on ST, year, region of collection and presence/absence of acquired *β*-lactamases. The ST164 lineage is highlighted to show its distinct clustering pattern.

Analysis of the 621 genomes identified 2,109 gene clusters in the core genome. Recombination masking was not applied to this dataset, as application of ClonalFrameML to the full multi-lineage dataset resulted in masking of ~96% of alignment sites, indicating misclassification of deep inter-lineage divergence as recombination. The maximum-likelihood phylogeny inferred directly from the core-genome alignment revealed that isolates belonging to the same ST and their locus variants [up to triple-locus variants (TLVs)] consistently formed monophyletic groups (Fig. 4). The tight clustering characterized by short-branch length highlights the clonal expansion with limited intra-lineage diversification. The largest cluster (red branch), majority corresponding to ST2, also included closely related DLV (ST129) and TLV (ST215) subclusters. These groups exhibited minimal divergence, reflecting that they shared a recent common ancestor and ongoing clonal dissemination. In contrast, several smaller clusters and singletons (black branches) displayed relatively longer branch lengths, suggesting greater genetic heterogeneity possibly shaped by horizontal gene transfer events or limited expansion from ancestral lineages.

Interestingly, the phylogeny exhibited a bifurcating structure near the root, delineating two major evolutionary trajectories. One lineage comprised a distinct cluster of ST164 and its SLVs, ST1131 and ST1479 (highlighted in blue), while the other encompassed the majority of isolates, including the dominant epidemic clones ST2, ST16, ST25 and ST374. This topology suggests early divergence of the ST164 lineage, potentially representing a separate evolutionary trajectory within the *A. baumannii* population in Thailand. Despite being the second most prevalent ST in the dataset, the members of ST164 lineage displayed markedly shorter overall branch lengths compared to other major lineages. The limited clustering of this lineage may also reflect limited diversification, restricted transmission or a relatively recent emergence rather than ongoing clonal expansion. Consistent with the phylogenomic observation, the goeBURST minimum spanning tree based on the MLST allelic profiles (Fig. 2b) also showed ST164 and its locus variants forming a separate clonal group, distinct from the main interconnected network of STs. In contrast, the majority of the STs displayed varying degrees of relatedness, with the exception of seven identified singletons (ST1, ST108, ST267, ST653, ST1057, ST1432 and ST1433), which were genetically distant from any established clonal complexes.

### Lineage-focused phylogenomic analysis of *A. baumannii* ST2 suggests microevolutionary divergence

**Fig. 5.**
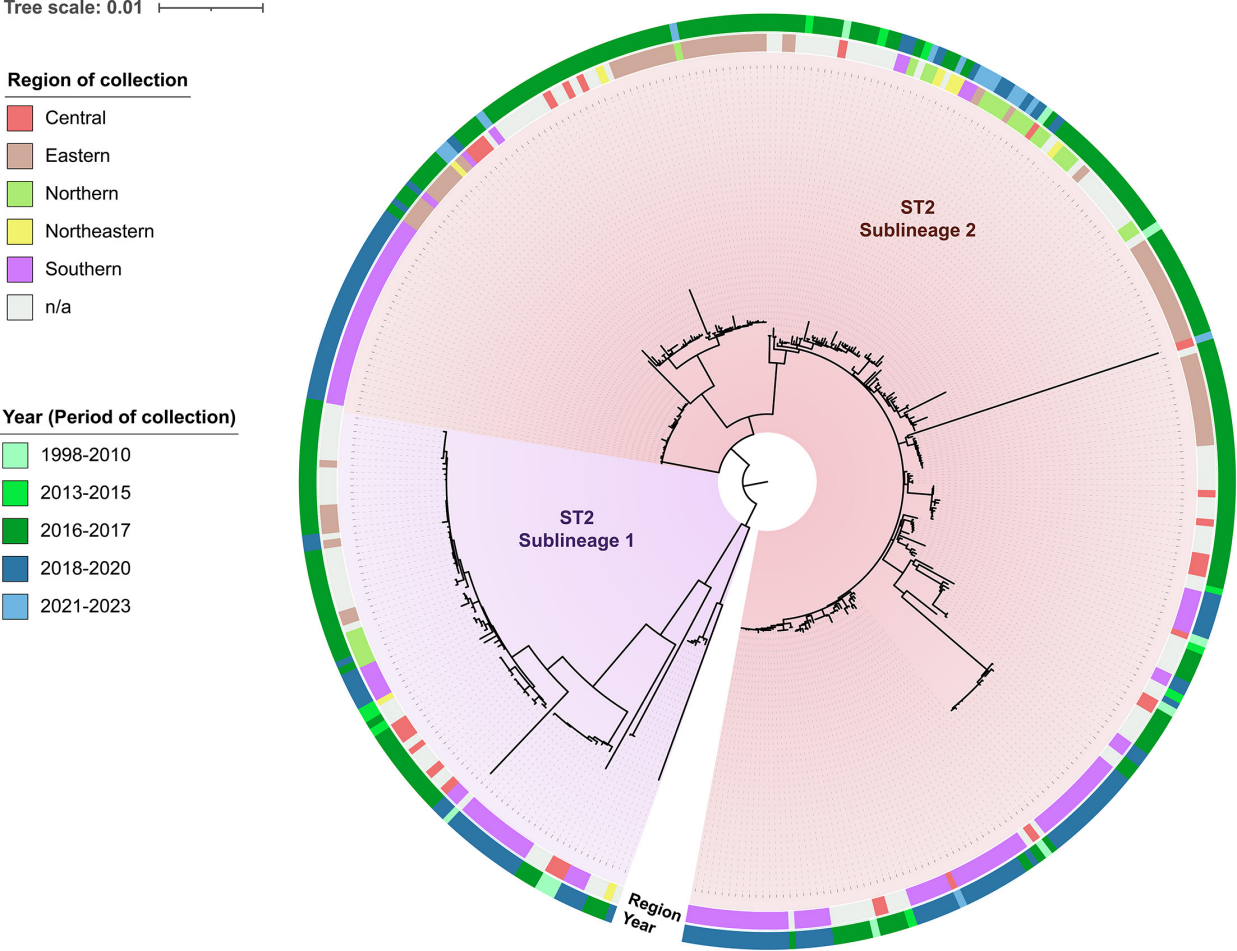
Maximum-likelihood phylogeny of 374 *A*. *baumannii* ST2 genomes originating from Thailand. The tree was inferred based on ClonalFrameML-masked-core genome SNPs across 2,736 gene clusters. Recombination-filtered SNPs were aligned and used for phylogenetic reconstruction in IQ-TREE2 under SYM+ASC+G4 substitution model with 1,000 ultrafast bootstrap replicates. The final tree was visualized in iTOL and displayed with unrooted topology. Concentric rings indicate metadata on the year and region of collection. The sublineage 1 and sublineage 2 are highlighted in purple and red, respectively.

A focused phylogenomic analysis was carried out to explore intra-lineage variation among ST2 isolates, the most prevalent clone in the dataset. The pangenome of this lineage comprised 2,736 gene clusters classified as the core genome from 374 ST2 genomes. A maximum-likelihood phylogeny reconstructed from ClonalFrameML-masked core-genome SNPs revealed evidence of microevolutionary divergence within this clonal lineage. Although ST2 genomes appeared as a tight cluster in the broader 621-genome phylogeny, finer resolution identified distinct divergence patterns within the lineage (Fig. 5).

The ST2 lineage split into two distinct sublineages, hereafter referred to as sublineage 1 and sublineage 2, which differed in overall branch length, with sublineage 1 exhibiting comparatively longer branches. The dataset was largely composed of isolates collected before 2018 (*n*=236), accounting for 61 and 56% of the genomes in sublineage 1 and sublineage 2, respectively. Genomic clustering also showed patterns of regional and temporal association, with closely related isolates from the same region or collection period frequently grouping together. Notable exceptions were observed, such as isolate MTC47026, collected from the central region during 2021–2023, which clustered among isolates predominantly collected from the southern region between 2018 and 2020. A subset of genomes, including SAMN14669787 (sublineage 1) and MTC47002 (sublineage 2), displayed longer branch lengths relative to other members of their closely related clusters.

Examination of the gene presence–absence matrix from the 650-genome dataset revealed patterns indicative of genes uniquely associated with the major clonal complex, particularly within ST2 lineage (Fig. S6). This prompted further investigation on the Roary matrix to identify ST2-specific genes. By applying a stringent filter of genes that are present in at least 90% of ST2 genomes but not more than 10% in non-ST2 genomes, a total of 16 gene clusters were identified (Table S5). The relatively conservative number of ST2-specific clusters likely reflects the stringent dual filtering criteria, where the threshold of less than 10% in non-ST2 genomes is particularly restrictive given the shared gene content between ST2 and its closely related SLVs/DLVs. Additionally, the default clustering threshold (95% amino acid identity) applied in pangenome reconstruction may collapse subtly divergent ST2-specific variants into shared clusters.

These clusters include the genes that encode proteins associated with diverse functional categories, including envelope biosynthesis, stress response and regulatory functions. Notably, GtrOC6 glycosyltransferase (*group_2897*) is linked to Lipooligosaccharide biosynthesis, suggesting potential structural modifications specific to ST2. Additional clusters include a polysaccharide deacetylase (*group_6527*, COG G) and a type-1 fimbrial protein (*smf-1_2*, COG NU), which may contribute to environmental persistence and surface adhesion. Genes associated with redox metabolism and stress adaptation were also identified, including glutathione S-transferase (*gst*, COG O) and NAD(P)-dependent oxidoreductase (*group_3845*, COG S). The presence of a pilus assembly protein (*pilA*) further suggests potential lineage-specific differences in motility or host cell adhesion.

### Bayesian inference reveals recent emergence and population dynamics of *A. baumannii* ST164 in Thailand

A similar lineage-specific phylogenomic approach was applied to the ST164 cluster, which comprised 61 genomes sharing 2,843 gene clusters in the core genome. Despite the increased resolution provided by lineage-specific SNP alignment, the resulting maximum-likelihood tree showed that ST164 and its SLV ST1479 remained tightly clustered within a well-supported monophyletic group (Fig. S7). Interestingly, two genomes belonging to ST1131 (SAMN40441921, SAMN28045321) formed a compact subcluster with very short branches near the root of the tree, diverging prior to the emergence of the main ST164 lineage.

Due to its unique occurrence as the second most prevalent ST in the pangenome dataset yet exhibiting limited diversity as inferred from the relatively short branch lengths, Bayesian temporal analysis was conducted to explore the evolutionary dynamics of the ST164 lineage over time in Thailand. Initial assessment of the temporal signal from the maximum-likelihood tree revealed a low *R*^2^ value of 0.013. However, re-rooting using the best-fitting root improved the *R*^2^ to 0.503, indicating a moderate temporal signal. BEAST analyses were subsequently conducted using three molecular clock models: strict clock, relaxed lognormal clock and random local clock. Model comparison based on Tracer-derived statistics suggested that both strict and relaxed lognormal clock achieved adequate convergence, with all ESSs exceeding 200 (Table S6). Notably, the relaxed lognormal clock model estimated a mean substitution rate of 4.44×10^−3^ substitutions per site per year, with a non-zero ucldStdev value (0.555) and coefficient of variation of 0.597, suggesting substantial rate variation among branches and thus supporting the suitability of the relaxed clock model. The random local clock model also achieved adequate convergence, estimating a mean of 1.5 rate changes across the tree, indicating insufficient rate heterogeneity to justify its additional complexity. Model selection by marginal likelihood estimation was inconclusive, as the strict clock returned an infinite value during path sampling, indicating instability in marginal likelihood estimation for that model. Therefore, final model selection was based on biological plausibility, model parameters and convergence diagnostics with the relaxed lognormal clock chosen for downstream analyses.

The resulting MCC tree (Fig. S8) revealed consistent temporal clustering and confirmed the clonal structure of the ST164 lineage in relation to sampling time. The uniformly short terminal branches further confirm the low genetic diversity of ST164 genomes within this dataset. The relaxed lognormal clock estimated that ST164 lineage originated ~65 years ago (tree height ~65 years), corresponding to an estimated time to the most recent ancestor (tMRCA) of ~1958. A major diversification event, however, occurred much more recently (between 2010 and 2020) as indicated by the Bayesian skyline plot (Fig. S9), which showed a decline in effective population size around 2010, followed by a sharp rebound between 2013 and 2015. The population size then plateaued and remained relatively stable through to the most recent sampling year (2023), indicating sustained endemic presence of this lineage rather than ongoing rapid expansion.

However, the estimated tMRCA of ~1958 and mean substitution rate of 4.44×10^−3^ substitutions per site per year appeared unexpectedly ancient and elevated for a recently emerged clone, suggesting possible residual distortion in the temporal estimates. Closer examination of the MCC tree (Fig. S8) revealed that the inclusion of ST1131, a SLV of ST164 but genetically divergent based on the maximum-likelihood phylogeny (Fig. S7), likely contributed to inflation of the tMRCA estimate through branch length extension. To better characterize the evolutionary dynamics of ST164, a separate analysis was performed excluding ST1131, retaining only ST164 (*n*=52) and its close SLV ST1479 (*n*=7). In this refined dataset, the temporal signal improved substantially (*R*^2^=0.517 with default root, 0.626 with best-fitting root), reflecting a stronger molecular clock signal following the removal of the divergent outgroup. The relaxed lognormal clock model estimated that ST164 emerged ~2013–2014 (tree height ~9 years), with a mean substitution rate of 1.09×10^−2^ substitutions per site per year. The Bayesian skyline plot for this refined dataset revealed a rapid expansion of effective population size coinciding with the estimated emergence of ST164 ~2014, followed by stabilization through to 2023 (Fig. S9). The wide 95% highest posterior density intervals, however, indicate considerable uncertainty in these demographic reconstructions, likely reflecting the limited genetic diversity and constrained sampling window of this recently emerged lineage.

**Fig. 6.**
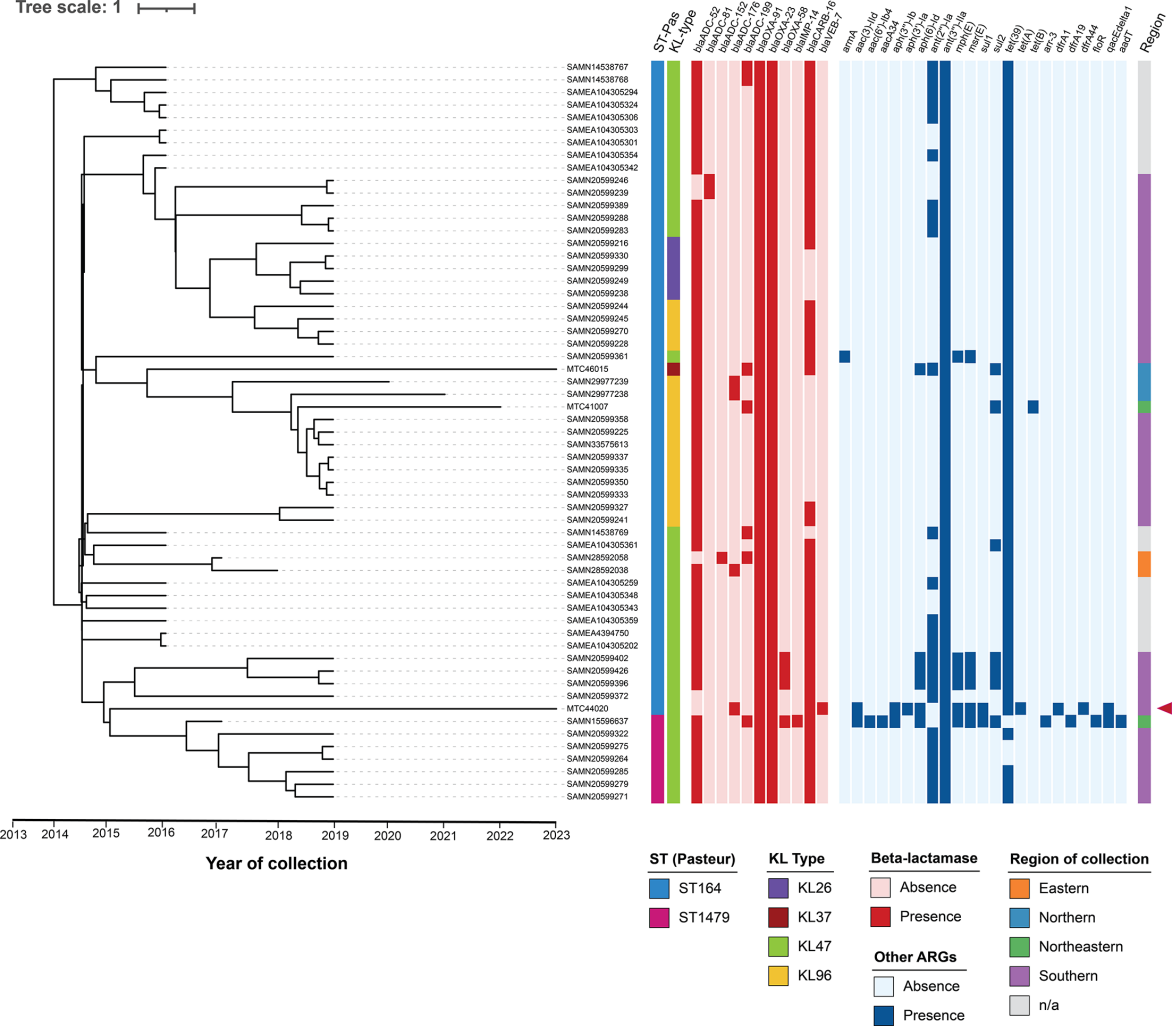
Time-calibrated MCC phylogenetic tree of ST164 lineage. The tree was generated using TreeAnnotator from the posterior distribution trees inferred by a relaxed lognormal clock model in BEAST. The x-axis indicates the time scale from the estimated tMRCA to the most recent sampling year. Branches were manually rotated in iTOL to improve readability. The tree is also annotated with metadata, including ST (Pasteur scheme), KL type, presence or absence of resistance genes and region of collection. The MTC44020 within the ST1479-associated cluster that carries a distinct resistome is marked by red arrowheads.

Notably, the MCC tree generated from the refined dataset (excluding ST1131) provided improved resolution of time-scaled phylogeny within ST164 cluster (Fig. 6). Excluding the divergent ST1131 genomes reduced the branch-length inflation, as reflected by a shorter tMRCA, enabling clearer differentiation among ST164 genomes that previously appeared as a tightly clustered monophyletic group. Capsule K locus (KL) typing further revealed that ST164 displayed limited KL diversity (four KL types, with KL47 comprising 62.7%), in contrast to greater KL diversity observed in ST2 (16 KL types). The outer core locus (OCL) type was highly conserved, with all of the genomes belonging to OCL5. The MCC tree also showed that five ST164 genomes clustered with ST1479. Within this group, several ST164 members harboured additional resistance determinants, most notably *blaOXA-58-like*. One isolate sequenced in this study (MTC44020) also fell within this subcluster but possessed a distinct resistome profile, including a different *blaADC* allele and acquisition of *blaVEB-7*, but not *blaOXA-58-like*.

## Discussion

Antimicrobial resistance in *A. baumannii* continues to impose a substantial burden on the healthcare system in Thailand, where carbapenem resistance rates are among the highest reported in the Asia-Pacific region [21]. Although multiple studies have described the predominance of a small number of high-risk clones, the genomic features underlying their persistence and diversification remain incompletely characterized. By consolidating newly sequenced clinical isolates with publicly available genomes, this study provides an updated genomic landscape of *A. baumannii* in Thailand, identifies lineage-associated resistome signatures and characterizes population structure and evolutionary trajectories of established and emerging lineages.

Among the intrinsic resistance determinants, *adeC* was highly prevalent in ST2 and ST215, but relatively rare in other lineages, resulting in an overall prevalence of ~70% across the dataset. Consistent with this, lower prevalence of *adeC* compared to other components of AdeABC has been reported elsewhere [54,55], although lineage-level resolution was not examined. The *adeC* gene encodes an outer membrane component of AdeABC efflux pump, a major multidrug efflux system in *A. baumannii* [56].

Previous studies have suggested that *adeC* is dispensable for AdeABC-mediated resistance, as an alternative outer membrane channel may compensate for its absence [57,58]. However, in the present dataset, *adeC* showed a striking enrichment in ST2 genomes. This pattern is notable given that all newly sequenced ST2 isolates exhibited XDR phenotypes, raising the possibility that retention of an intact AdeABC operon may confer a selective advantage in this lineage. Supporting this interpretation, a prior study demonstrated that the *adeC*-positive isolates exhibit higher resistance to multiple antimicrobials, including carbapenems and netilmicin [55]. Together, these observations suggest that *adeC* may serve not only as a functional contributor to resistance but also as a lineage-associated marker of high-risk clones.

The allelic diversity of intrinsic *β*-lactamases further highlights the lineage-structured nature of the Thai *A. baumannii* population. Distinct ST-specific combinations of *blaADC/blaOXA-51-like* allelic profile were consistently observed, indicating long-term maintenance of these allelic profiles within clonal backgrounds. The ST-specific distribution of these intrinsic *β*-lactamase alleles may also reflect underlying geographical trends. In line with this, a recent global study analysing over 30,000 *Acinetobacter* genomes reported *blaADC-73* and *blaOXA-66* as the most prevalent alleles from each group and lineage association observed overall [59]. In contrast, a study of 94 CRAB isolates from US hospitals identified *blaOXA-66* as the most common among ST2 isolates but found *blaADC-212* to be the most predominant *blaADC* variants, and *blaADC-73* was not detected [60]. In the present study, the predominance of *blaADC-73*/*blaOXA-66* in ST2 may therefore represent a characteristic feature of Thai CRAB isolates, with a similar principle potentially applying to other STs. These findings notably imply that the intrinsic *β*-lactamase allele combination may carry geographical signal and could serve as useful auxiliary markers for tracking regional lineage dynamics.

In the context of acquired *β*-lactamase genes, the *blaOXA-23-like* carbapenemase was the most prevalent across 650 genomes, in agreement with previous reports [13,59,61]. Diversity of *β*-lactamase genes acquisition appeared more prevalent in non-ST2 genomes, supporting the notion that ST2 tends to maintain a stabilized resistome, as initially observed among 38 sequenced isolates. This further suggests that the dominant ST2 lineage is sustained by clonal expansion rather than extensive horizontal acquisition of resistance determinants. Several other acquired *β*-lactamase displayed ST-specific patterns despite lower overall frequencies, including *blaTEM*, *blaVEB*, *blaNDM*, *blaOXA-58-like* and *blaCARB*. Among these, *blaNDM* was notable for its lineages-stratified allelic distribution.

Two *blaNDM* variants were identified (*blaNDM-1* and *blaNDM-5*). Although *blaNDM* was slightly more common in ST16 (*n*=22) than in ST2 (*n*=17), the allele distribution was markedly different. All ST16 genomes harboured *blaNDM-1*, whereas *blaNDM-5* was detected only in ST2, accounting for approximately half of the *blaNDM*-positive ST2 genomes. The *blaNDM-5* has been reported to exhibit higher hydrolytic activity against carbapenems and cephalosporins than *blaNDM-1* [62,63] and is increasingly associated with plasmid-mediated dissemination [64,66]. In Thailand, *blaNDM-5* has been documented in XDR and MDR ST2 CRAB isolates from both clinical and hospital environmental sources, potentially in association with class I integrons [26]. Notably, *blaNDM-5* has been linked to reduced susceptibility to cefiderocol [67,68], suggesting potential clinical implications if dissemination continues, especially in settings where cefiderocol use remains limited.

Beyond resistome signatures, distinct phylogenetic placement was notably observed among dominant lineages in the dataset. In particular, the ST164 genomes and its associated locus variants consistently formed a separate cluster from other major epidemic lineages, including ST2, ST16, ST25 and ST374. This separation was consistently observed in both the goeBURST minimum-spanning tree and the maximum-likelihood core-genome SNP phylogeny, indicating an independent evolutionary trajectory.

ST164 is of particular epidemiological relevance, having recently been designated IC11 following increasing reports of global dissemination [15]. In the present dataset, ST164 appeared as the second most prevalent lineage, accounting for ~10% of the analysed genomes, in agreement with previous surveillance reports from Thailand [22,23]. Comparable prevalence has been reported in neighbouring Malaysia (5–10%) [69,70]. In China, reported prevalence has ranged from 8% in a tertiary hospital [71] to a rapid expansion reaching 49.2% in 2021, coinciding with a decline in ST2 [72]. Outside Asia, ST164 has been detected in Europe, including Denmark, where the cases were linked to travel histories to Thailand, Iran and Mauritius [15]. Additional reports have documented the widespread occurrence of this lineage, including Vietnam, Egypt, Germany, Kuwait and Nepal [73,77].

Public database records further indicate that ST164 has circulated internationally for some time, with earliest isolation years recorded in Pathogenwatch (2006, USA) and PubMLST (2002, Brazil). In contrast, both databases report the earliest record of an isolate from Thailand as dating to 2016, suggesting a more recent establishment of this lineage in the Thai clinical setting. Bayesian phylodynamic analyses of ST164 genomes in the present study further suggest that this lineage emerged in Thailand relatively recently, ~2013–2014, and has since maintained a stable effective population size rather than undergoing rapid expansion.

Several additional features provide insights into the epidemiological relevance of ST164. The limited capsule locus diversity observed within ST164, together with its conserved OCL type, supports the notion of recent emergence and restricted diversification. Nevertheless, the identification of ST164 subclusters carrying additional resistance determinants, including *blaOXA-58-like*, indicates ongoing acquisition events that may enhance its adaptive potential. Continued circulation under antimicrobial pressure could therefore facilitate further diversification of this lineage, underscoring the importance of longitudinal genomic surveillance to detect early signals of expansion or phenotypic shifts. Recent studies in China reported rapid expansion of ST164 driven by co-occurrence of *blaOXA-23* and *blaNDM-1* [71,72], although this pattern has not yet been observed in Thailand.

This study has several limitations. Reliance on public genomes may contribute to underrepresentation of less prevalent STs and influence resistome estimates. Metadata such as antimicrobial susceptibility results, year of isolation and geographic origin were also inconsistently available, limiting more granular epidemiological inference. For the sequenced isolates, quantitative MIC data were unavailable for the majority of isolates, as susceptibility testing at participating hospitals was reported as interpretive categories (S/I/R) and laboratory confirmation relied on disc diffusion (except for colistin), precluding systematic gene-phenotype correlation analyses.

Interpretation of the temporal dynamics of ST164 is further constrained by the high estimated mean substitution rate ($1.09\times 10^{-2}$ substitutions per site per year), which is substantially above the rate reported for IC1 and IC2 lineages. Previous studies have reported rates for GC1 and GC2 in the range of approximately $10^{-6}$ to $10^{-8}$ substitutions per site per year [2,16, 78, 79], whereas no such estimates exist for ST164 to our knowledge. This likely reflects the recent emergence and limited genetic diversity of ST164, which is linked to the well-established time dependency of molecular rate estimates, wherein rates calculated over short evolutionary intervals tend to be inflated due to the presence of transient or deleterious mutations that have not yet been purged by purifying selection [80,81]. Given the short sampling window (2015–2023) and limited diversity of ST164 in this dataset, it is probable that the estimated rate reflects such time-dependent bias. Broader temporal sampling and incorporation of global datasets will be essential to clarify and refine the evolutionary trajectory of ST164 and assess its future dissemination.

Taken together, this study demonstrates that the CRAB population in Thailand is structured around a limited number of dominant lineages with distinct genomic and resistome characteristics. ST2 remains the most prevalent clone and is consistently associated with specific intrinsic resistance signatures, including the presence of *adeC* and characteristic *blaADC/blaOXA-51-like* allelic combinations. In contrast, ST164 represents an emerging lineage with a clearly distinct phylogenetic placement, limited genetic diversification and evidence consistent with relatively recent establishment in the Thai clinical settings.

## Supplementary material

10.1099/mgen.0.001716 Uncited Supplementary Material 1.

10.1099/mgen.0.001716 Uncited Supplementary Material 2.

## Abbreviations

AME: aminoglycoside-modifying enzyme
ANI: average nucleotide identity
CRAB: carbapenem-resistant *Acinetobacter baumannii*
DLV: double-locus variant
ESS: effective sample size
IC11: international clone 11
KL: K locus
MCC: maximum clade credibility
MCMC: Markov chain Monte Carlo
MDR: multidrug-resistant
MLST: multi-locus sequence typing
OCL: outer core locus
SLV: single-locus variant
ST: sequence type
TLV: triple-locus variant
tMRCA: time to the most recent ancestor
XDR: extensively drug-resistant
