# Supplementary material for: Genomic signatures of dominant clones and evolutionary divergence of Acinetobacter baumannii in Thailand
Source: Microb Genom. 2026 May 14;12(5):001716. doi: 10.1099/mgen.0.001716 (PMC13175534; doi:10.1099/mgen.0.001716)
Supplement: Uncited Supplementary Material 1. [file mgen-12-01716-s001.pdf]

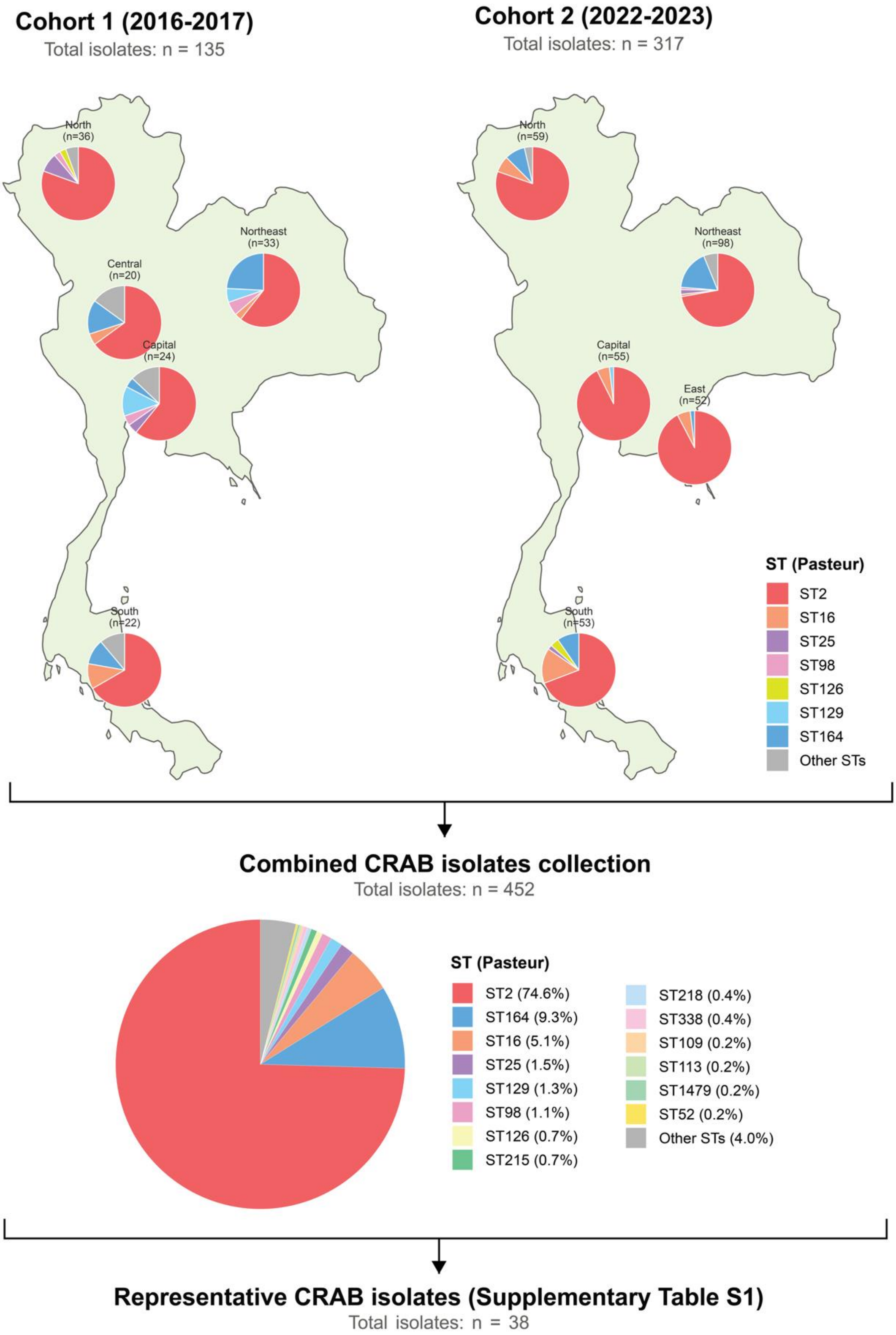

Supplementary Figure S1. Lineage distribution of 452 CRAB isolates collected from two previous independent surveillance cohorts. These isolates served as the basis for selection of 38 representative isolates for WGS in this study.

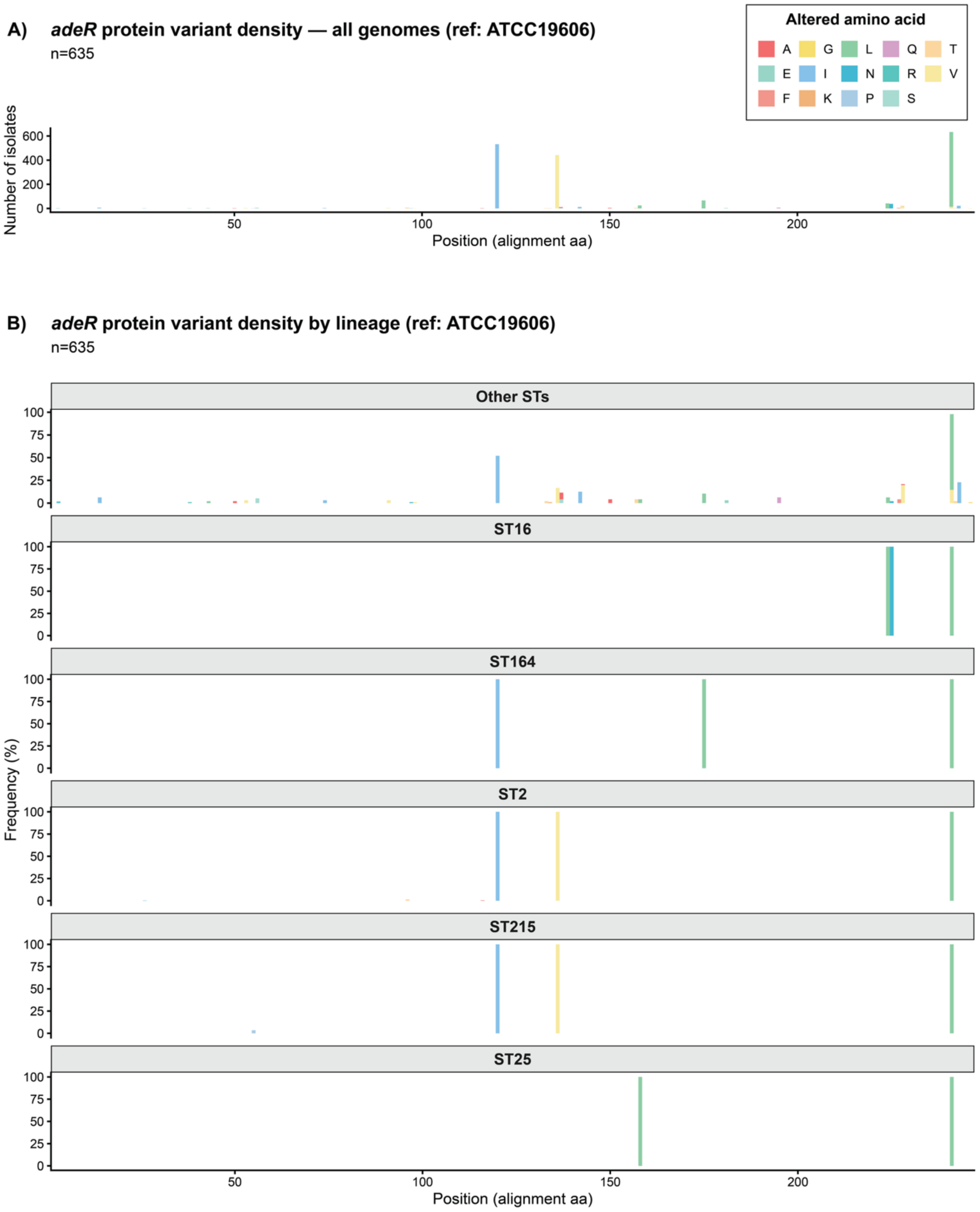

Supplementary Figure S2. Non-synonymous SNP density of *adeR* across 650 genomes (A) and by lineage (B). The *adeR* protein sequence of *A. baumannii* ATCC 19606 (GenBank Accession CP045110.1) was used as the reference for BLASTn analysis. Panel A shows the number of isolates carrying each amino acid substitution per alignment position. Panel B shows the frequency (%) of each substitution within each sequence type. Bars are coloured by the altered amino acid identity.

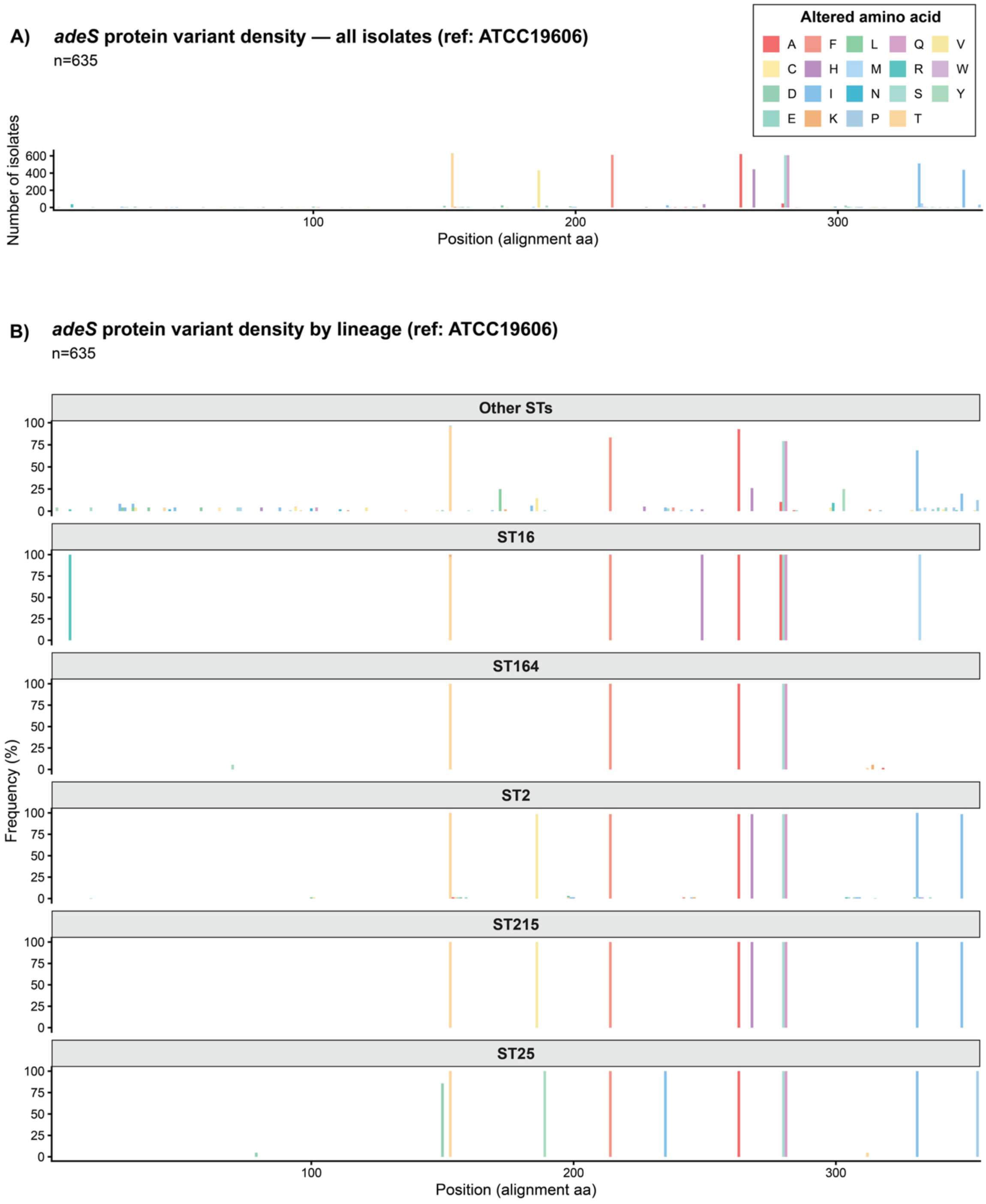

Supplementary Figure S3. Non-synonymous SNP density of *adeS* across 650 genomes (A) and by lineage (B). The *adeS* protein sequence of *A. baumannii* ATCC 19606 (GenBank Accession CP045110.1) was used as the reference for BLASTn analysis. Panel A shows the number of isolates carrying each amino acid substitution per alignment position. Panel B shows the frequency (%) of each substitution within each sequence type. Bars are coloured by the altered amino acid identity.

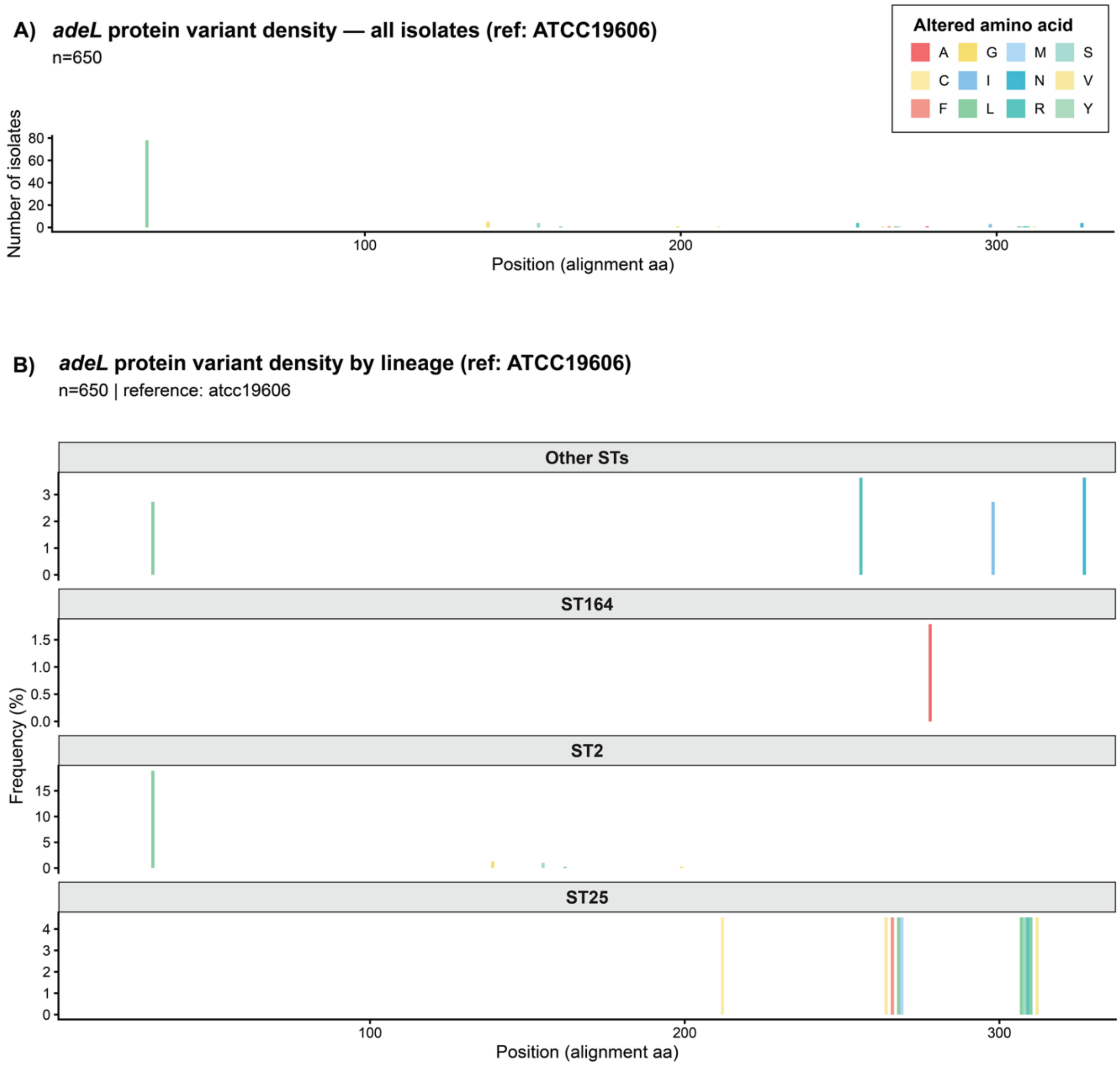

Supplementary Figure S4. Non-synonymous SNP density of *adeL* across 650 genomes (A) and by lineage (B). The *adeL* protein sequence of *A. baumannii* ATCC 19606 (GenBank Accession CP045110.1) was used as the reference for BLASTn analysis. Panel A shows the number of isolates carrying each amino acid substitution per alignment position. Panel B shows the frequency (%) of each substitution within each sequence type. Bars are coloured by the altered amino acid identity.

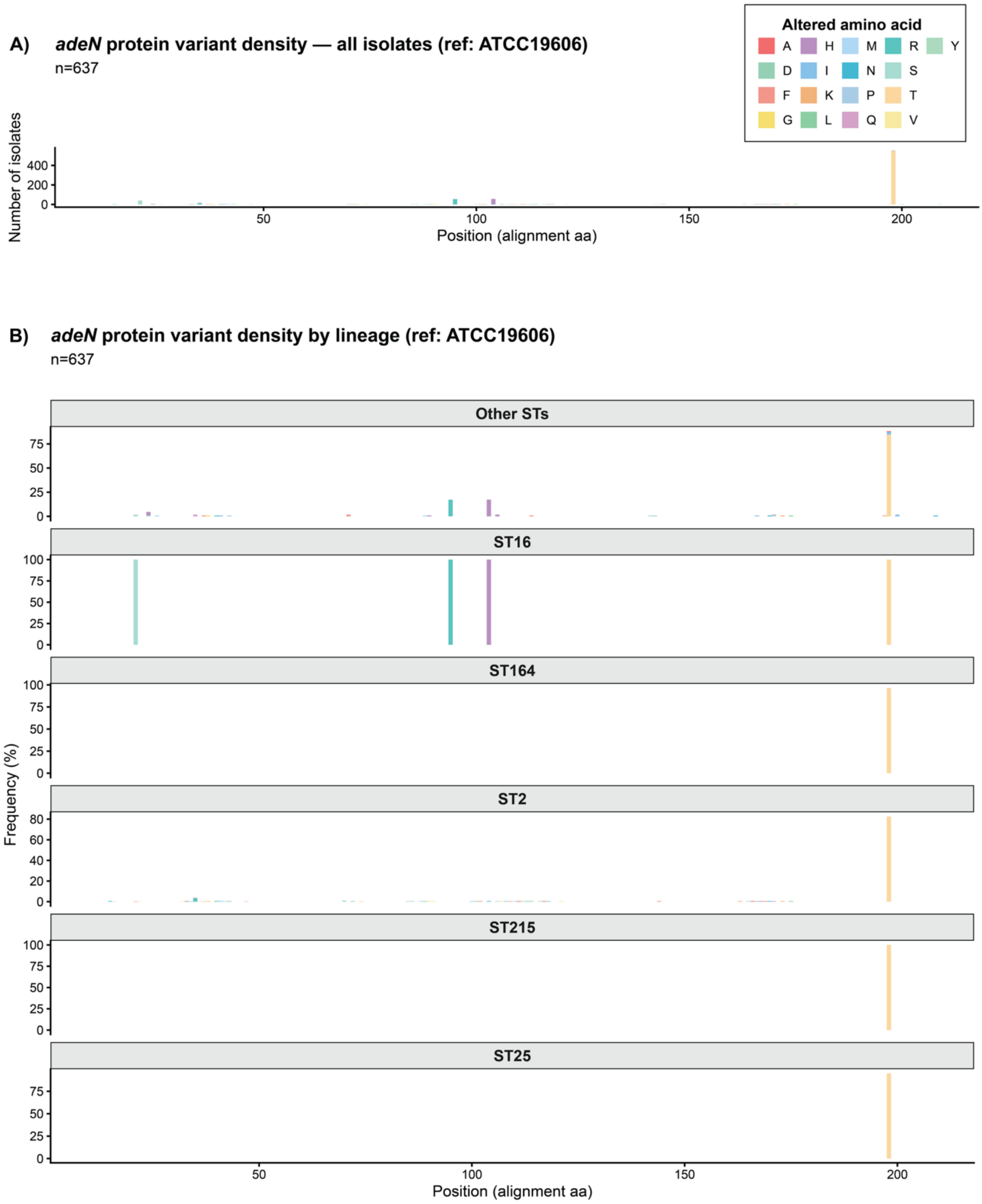

Supplementary Figure S5. Non-synonymous SNP density of *adeN* across 650 genomes (A) and by lineage (B). The *adeN* protein sequence of *A. baumannii* ATCC 19606 (GenBank Accession CP045110.1) was used as the reference for BLASTn analysis. Panel A shows the number of isolates carrying each amino acid substitution per alignment position. Panel B shows the frequency (%) of each substitution within each sequence type. Bars are coloured by the altered amino acid identity.

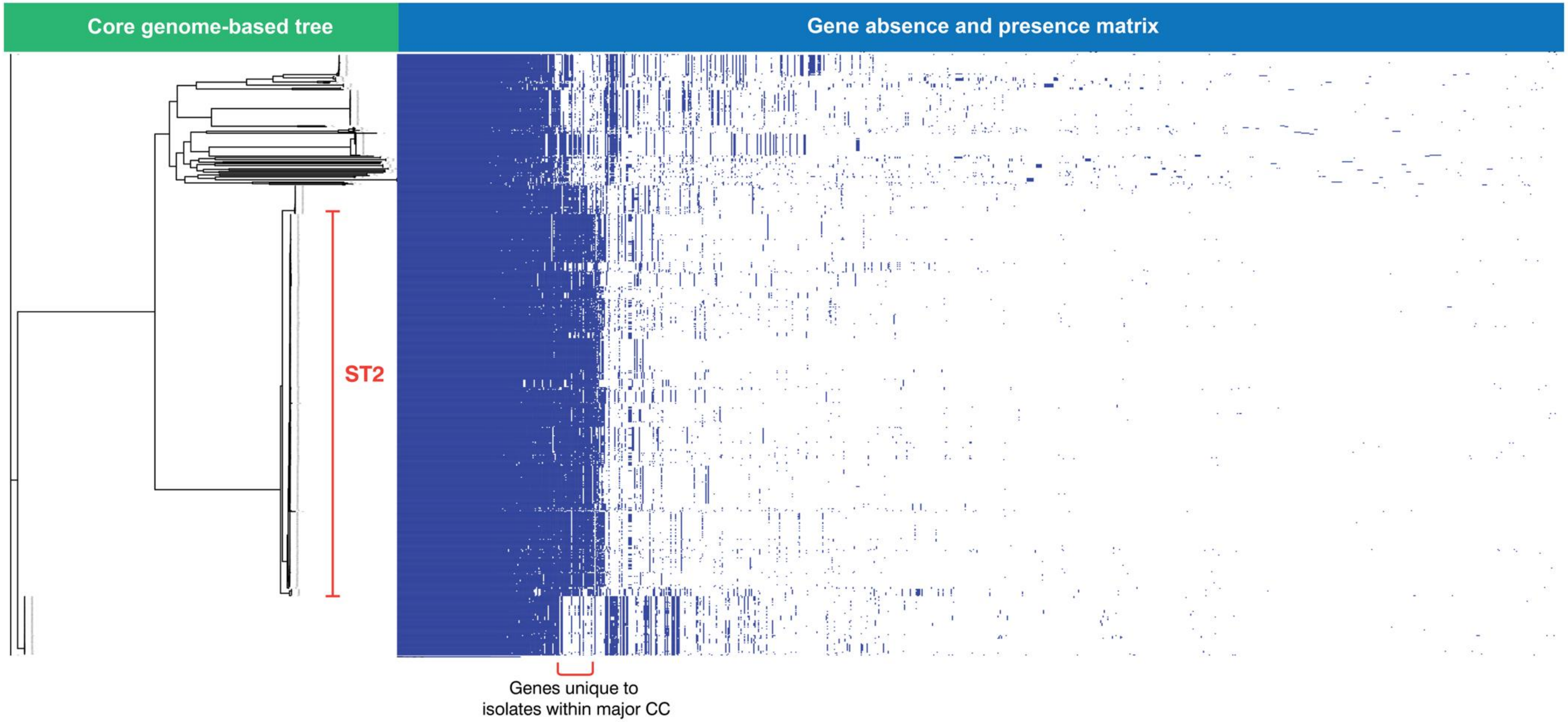

Supplementary Figure S6. Phylogenetic tree of 650 *A. baumannii* genomes with gene absence and presence matrix. Figure was visualized in Phandango webserver (https://jameshadfield.github.io/phandango/). The phylogenetic tree is based on core genome alignment, and the gene absence-presence matrix represents a total of 19,903 gene clusters identified in the pangenome.

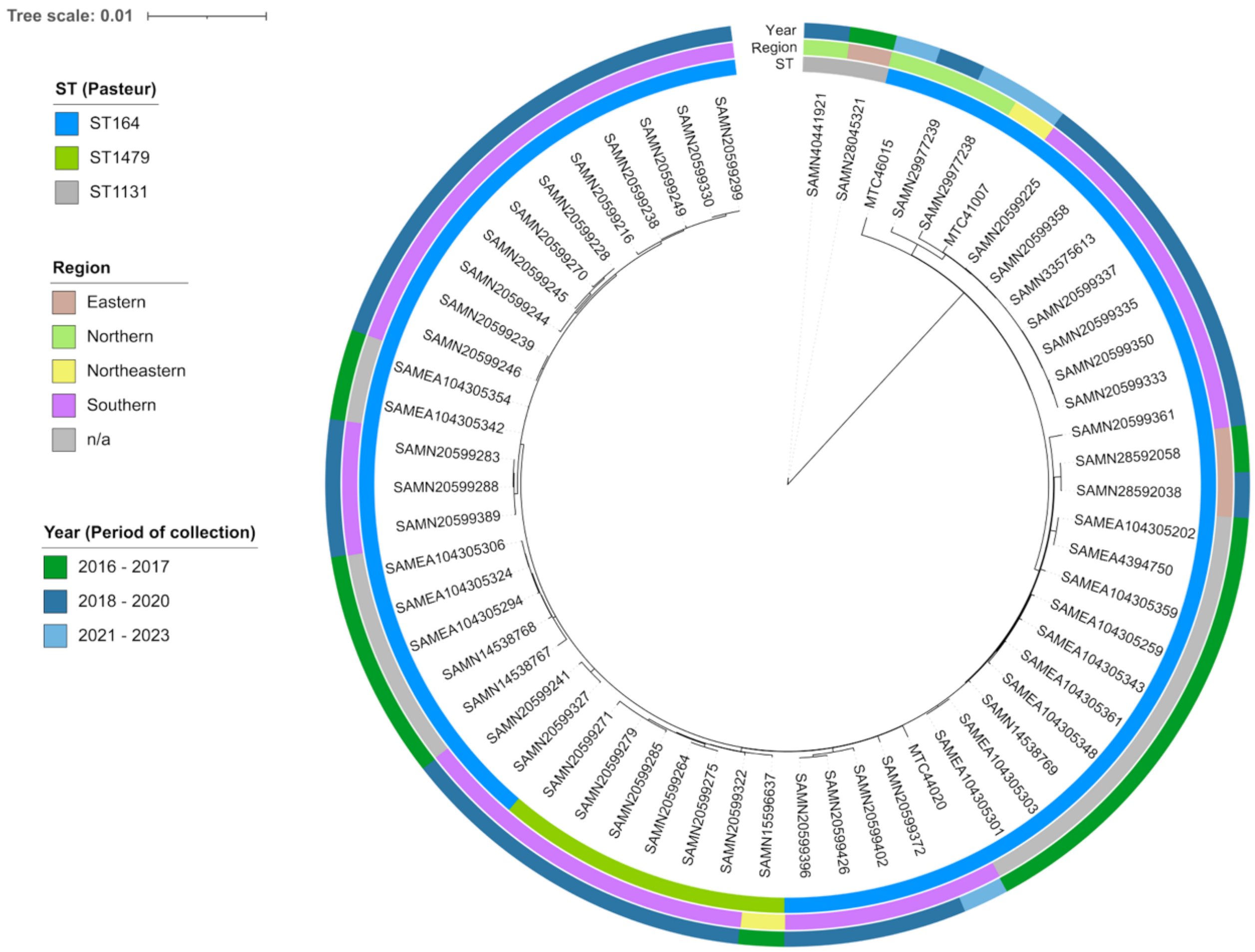

Supplementary Figure S7. Maximum-likelihood phylogeny of 61 *A. baumannii* genomes from Thailand belonging to the ST164 cluster. The tree was inferred from core genome SNPs across 2,843 gene clusters. Recombination-filtered SNPs were aligned and used for phylogenetic reconstruction in IQ-TREE2 under TVMe+ASC substitution model with 1000 ultrafast bootstrap replicates. The final tree was visualized in iTOL and displayed with unrooted topology. Concentric rings indicate metadata on ST, year and region of collection.

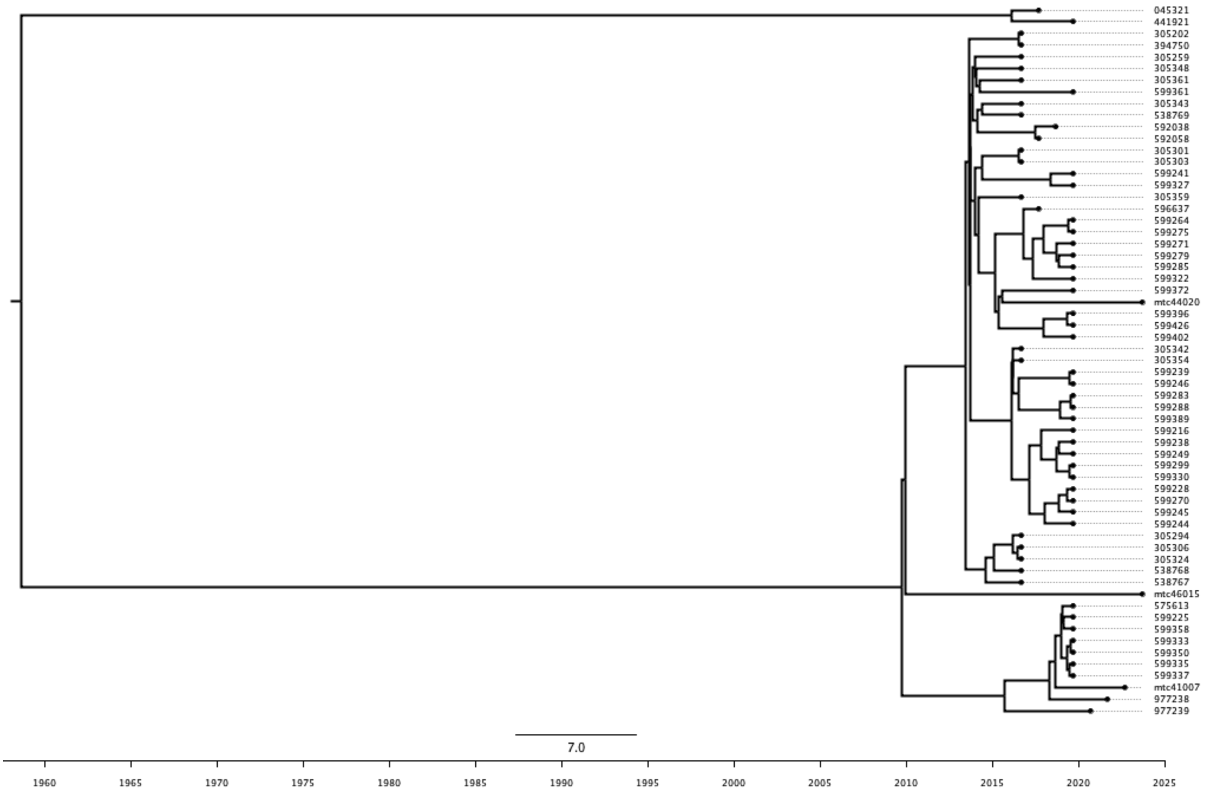

Supplementary Figure S8. Maximum clade credibility (MCC) tree of ST164 lineage. The tree was inferred from the posterior distribution trees generated by BEAST under a relaxed lognormal molecular clock model and summarized in TreeAnnotator. Tip labels are annotated with the year of isolation and the BioSample Accession. The tree was visualized in Figtree v.1.4.4 and scaled in units of calendar year.

A)

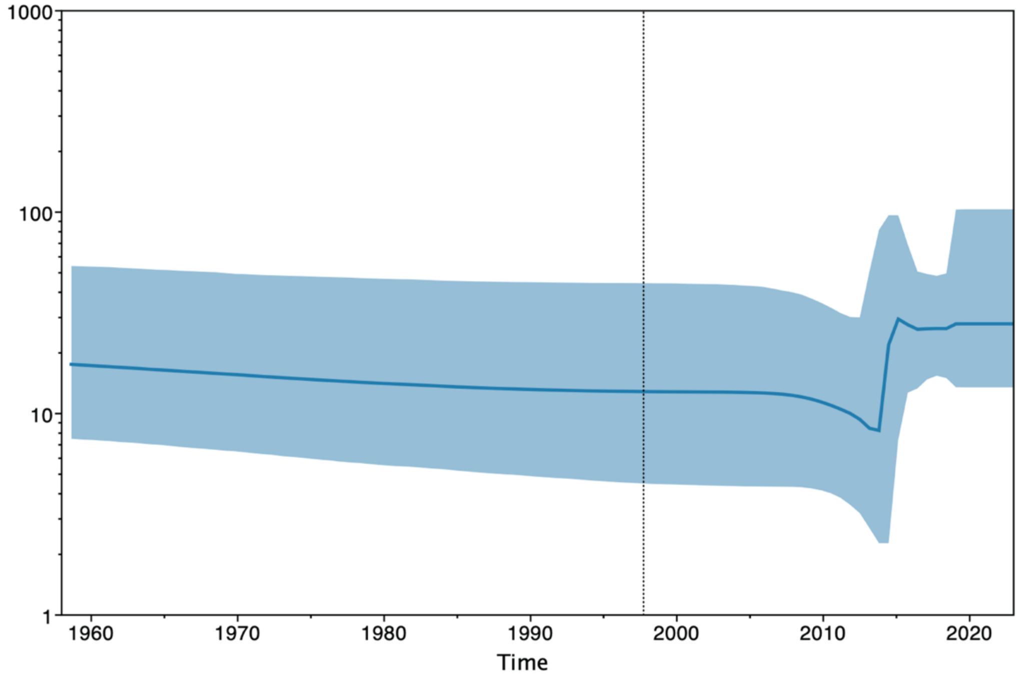

B)

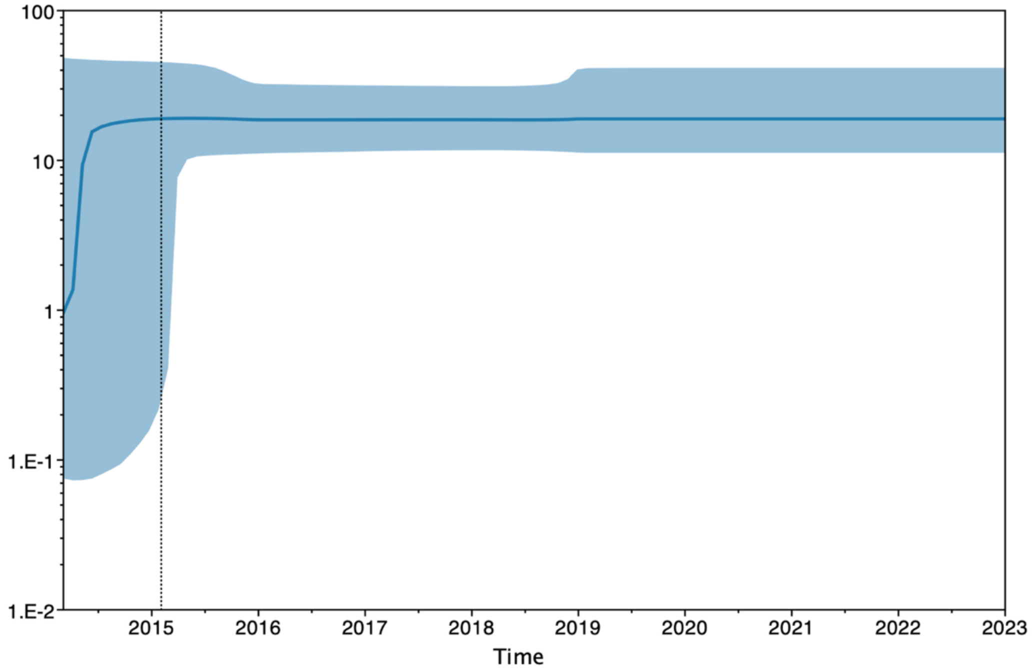

Supplementary Figure S9. Bayesian skyline plot of *A. baumannii* ST164 lineage in Thailand. (A) Effective population size (y-axis, log scale) of the ST164 lineage, including single-locus variants (SLVs) ST1131 and St1479, over time (x-axis). (B) ST164 lineage excluding the distantly related SLV1131. Both plots were generated using a relaxed lognormal molecular clock model in BEAST. The shaded area represents the 95% highest posterior density (HPD) interval.
